# Blood Pressure in Adolescence and Atherosclerosis in Middle Age

**DOI:** 10.1001/jamacardio.2025.4271

**Published:** 2025-11-19

**Authors:** Ángel Herraiz-Adillo, Hampus Eriksson, Viktor H. Ahlqvist, Marcel Ballin, Patrik Wennberg, Bledar Daka, Cecilia Lenander, Daniel Berglind, Carl Johan Östgren, Oskar Lundgren, Karin Rådholm, Pontus Henriksson

**Affiliations:** 1 Department of Health, Medicine and Caring Sciences, Linköping University, Linköping, Sweden; 2 Department of Research in Kalmar, Region Kalmar County, Kalmar, Sweden; 3 Department of Biomedicine, Aarhus University, Aarhus, Denmark; 4 Institute of Environmental Medicine, Karolinska Institutet, Stockholm, Sweden; 5 Clinical Geriatrics, Department of Public Health and Caring Sciences, Uppsala University, Uppsala, Sweden; 6 Department of Public Health and Clinical Medicine, Umeå University, Umeå, Sweden; 7 Institute of Medicine, School of Public Health and Community Medicine, Sahlgrenska Academy, University of Gothenburg, Gothenburg, Sweden; 8 Department of Clinical Sciences in Malmö, Centre for Primary Health Care Research, Lund University, Lund, Sweden; 9 Department of Global Public Health, Karolinska Institutet, Stockholm, Sweden; 10 Centre for Epidemiology and Community Medicine, Region Stockholm, Stockholm, Sweden; 11 Center for Wellbeing, Welfare and Happiness, Stockholm School of Economics, Stockholm, Sweden; 12 Centre of Medical Image Science and Visualization, Linköping University, Linköping, Sweden; 13 Crown Princess Victoria Children’s Hospital, Linköping University Hospital, Linköping, Sweden; 14 Division of Pediatrics, Department of Biomedical and Clinical Sciences, Linköping University, Linköping, Sweden; 15 Primary Health Care Center, Kärna, Sweden; 16 The George Institute for Global Health, University of New South Wales, Sydney, New South Wales, Australia

## Abstract

**Importance:**

Elevated blood pressure (BP) in adolescence has been linked to higher risk of cardiovascular disease mortality, as well as surrogate markers of atherosclerosis, such as carotid intima-media thickness and coronary artery calcification. However, these markers do not fully capture the complex spectrum of subclinical atherosclerotic cardiovascular disease.

**Objective:**

To examine the association between systolic and diastolic BP in adolescence and atherosclerosis in middle age, measured by coronary computed tomography angiography (CCTA).

**Design, Setting, and Participants:**

This population-based cohort study conducted in Sweden linked BP data from the Swedish Military Conscription Register (1972-1987) during adolescence to atherosclerosis data from the Swedish Cardiopulmonary Bioimage Study (2013-2018) during middle age. Data analyses were performed in May 2025.

**Exposure:**

Adolescent BP was categorized according to the 2025 American College of Cardiology/American Heart Association (ACC/AHA) and the 2024 European Society of Cardiology (ESC) guidelines.

**Main Outcomes and Measures:**

The primary outcome was coronary atherosclerosis, evaluated via CCTA stenosis. The associations were analyzed using multinomial logistic regression, adjusted (marginal) prevalences, and restricted cubic splines.

**Results:**

A total of 10 222 men with mean (SD) age of 18.3 (0.5) years at baseline and median (IQR) age of 57.8 (53.4-61.2) years at follow-up were included. At baseline, mean (SD) systolic BP (SBP) and diastolic BP (DBP) were 127.6 (10.7) mm Hg and 68.3 (9.5) mm Hg, respectively. After a median (IQR) follow-up of 39.5 (35.2-42.8) years, 4159 participants (45.7%) had 1% to 49% coronary stenosis and 784 (8.6%) had 50% or greater coronary stenosis. Elevated BP in adolescence was associated with coronary stenosis in a dose-response fashion. Adolescents with stage 2 hypertension had a higher risk of severe coronary stenosis (≥50%), with an odds ratio of 1.84 (95% CI, 1.40-2.42) and an adjusted prevalence of 10.1% (95% CI, 8.6%-11.5%) compared to those with normal BP (adjusted prevalence, 6.9%; 95% CI, 5.7%-8.1%). Elevated BP categories according to the 2025 ACC/AHA (120-129/<80 mm Hg) and the 2024 ESC (120-139/70-89 mm Hg) were associated with severe coronary atherosclerosis in middle age. The association was stronger for SBP than for DBP.

**Conclusions and Relevance:**

In this population-based cohort study, higher BP levels in adolescence were associated with a dose-dependent higher risk for atherosclerosis in middle age, particularly for severe coronary atherosclerosis. Excess risks of atherosclerosis were even evident in the elevated BP range in adolescence as defined by the 2025 ACC/AHA and 2024 ESC BP guidelines.

## Key Points

**Question:**

What is the association between blood pressure (BP) in adolescence and computed tomography–detected atherosclerosis in middle age?

**Findings:**

In this population-based cohort study of 10 222 men enrolled in the Swedish Military Conscription Register followed up for a median (IQR) duration of 39.5 (35.2-42.8) years, higher BP levels in adolescence were associated with greater risk of coronary atherosclerosis in middle age. Elevated risk was observed at BP of 120/80 mm Hg or higher.

**Meaning:**

These findings suggest that BP in adolescence plays a significant role in the development of coronary atherosclerosis in middle age, highlighting the importance of early-life focus on BP prevention strategies.

## Teaser

This population-based cohort study conducted in Sweden examines the association between systolic and diastolic blood pressure in adolescence and atherosclerosis in middle age.

## Introduction

High blood pressure (BP) is a leading global risk factor for premature death,^1,2^ accounting for about 10.8 million deaths in 2021. Although prevention and management of hypertension have largely focused on adulthood, hypertension can begin in childhood and adolescence,^3^ and its prevalence may be increasing due to rising obesity rates.^4,5^ Notably, BP moderately tracks from childhood to adulthood due to gene-environment interactions,^6,7^ underscoring the connection between early-life BP and adult cardiovascular disease (CVD) risk.

Several studies have linked elevated BP early in life with increased risk of CVD^8,9,10,11,12,13^ and CVD mortality in adulthood.^11,13,14^ Investigating the association between early-life BP and subclinical atherosclerosis, the etiological mechanism underlying most CVD, could provide valuable insights into the long-term consequences of early BP elevation. Prior research has associated elevated childhood and adolescent BP with surrogate markers of vascular damage in adulthood, such as increased carotid intima-media thickness,^15,16,17^ pulse wave velocity,^16^ or impaired endothelial function.^18^ In addition to these surrogate measures, 1 relatively small study involving 589 adolescents identified an association between elevated BP and coronary artery calcification (CAC), an imaging-based marker of atherosclerosis.^19^

However, to our knowledge, no study has used coronary computed tomography angiography (CCTA), an imaging modality that surpasses CAC by enabling direct visualization of the coronary arteries, assessment of stenosis, and detection of calcified, noncalcified, and mixed plaques. These noncalcified and mixed lesions are considered particularly high risk, as they are more prone to rupture and trigger cardiovascular events.^20,21^ Investigating the long-term links between early-life BP and CCTA-assessed atherosclerosis is essential to bridge the gap between adolescent BP elevation and adult CVD risk, as it provides mechanistic insight into disease development and identifies opportunities for prevention before clinical outcomes, such as CVD events or mortality, occur. Anatomical assessment provided by CCTA directly contributes to the emerging paradigm in coronary artery disease that prioritizes atherosclerotic burden over ischemia-focused functional assessment.^22,23^

Until 2024, the 2017 American College of Cardiology/American Heart Association (ACC/AHA) guidelines and the 2018 European Society of Cardiology (ESC) BP guidelines differed: the 2017 ACC/AHA guidelines defined elevated systolic BP (SBP) at 120 to 129 mm Hg,^24^ while the 2018 ESC guidelines considered this range as normal.^25^ The updated 2024 ESC guidelines now align more closely with the ACC/AHA guidelines, categorizing SBP of 120 to 139 mm Hg as elevated.^26^ Likewise, the newly released 2025 ACC/AHA guidelines reaffirm the 2017 thresholds.^27^ To date, no research has examined these BP thresholds in adolescence in relation to atherosclerosis in middle age using CCTA. Thus, this study aims to investigate the association between adolescent BP and coronary (CCTA-defined stenosis and CAC) and carotid (ultrasound-diagnosed plaque) atherosclerosis nearly 40 years after adolescent assessment.

## Methods

This population-based cohort study linked data from adolescents enrolled in the Swedish Military Conscription Register^28^ to data from the Swedish Cardiopulmonary Bioimage Study (SCAPIS)^29^ using personal identification numbers. Conscription data (1972-1987) included 82% to 92% of Swedish men, typically assessed at approximately 18 years of age. SCAPIS (2013-2018) is a population-based cohort of more than 30 000 adults aged 50 to 64 years recruited across 6 university hospitals in Sweden.

Eligible participants were men younger than 20 years at conscription with complete data on BP and covariates at baseline and atherosclerosis outcomes and covariates available at follow-up in SCAPIS. This study follows the Strengthening the Reporting of Observational Studies in Epidemiology (STROBE) reporting guidelines.^30^

eFigure 1 in Supplement 1 outlines the study flow. Of the 14 646 male participants in SCAPIS, 10 802 had matching conscription data. After exclusions, final sample sizes were 9110 participants (84.3%) for CCTA stenosis, 8925 (82.6%) for CAC score, and 10 205 (94.5%) for carotid plaque analysis.

Ethical approval was granted by the Swedish Ethical Review Authority (reference numbers 2021-06408-01 and 2022-04375-02), and all participants gave written informed consent. Additional details on data collection and imaging protocols are provided in the eMethods in Supplement 1.

### BP in Adolescence

BP at conscription was measured following a standardized protocol, with participants resting in a supine position for 5 to 10 minutes prior to BP measurement by auscultation.

BP was categorized according to the 2025 ACC/AHA BP guidelines^27^ into 4 mutually exclusive categories without separating SBP and diastolic BP (DBP) phenotypes: normal BP (SBP <120 mm Hg and DBP <80 mm Hg), elevated BP (SBP = 120-129 mm Hg and DBP <80 mm Hg), hypertension stage 1 (SBP = 130-139 mm Hg or DBP = 80-89 mm Hg), and hypertension stage 2 (SBP ≥140 mm Hg or DBP ≥90 mm Hg).

In addition, BP was categorized according to the 2024 ESC BP guidelines^26^ (eMethods in Supplement 1).

### Atherosclerosis in Middle Age

#### Coronary Atherosclerosis

The imaging protocol for SCAPIS has been previously published.^29^ Imaging was performed using a dual-source CT scanner with a Stellar Detector (SOMATOM Definition Flash [Siemens]). Contrast-enhanced CCTA (100-120 kV) and noncontrast electrocardiogram-gated CT (120 kV) were used to assess coronary stenosis and CAC, respectively, with analysis performed using the syngo.via software (Siemens).

For primary analysis, CCTA stenosis was defined based on the segment with the highest degree of stenosis among the 11 key coronary segments^31^ and categorized as no stenosis, 1% to 49% stenosis, and 50% or greater stenosis.^32^ Additionally, for linear splines and binomial analysis, a segment involvement score was calculated based on the total number of coronary segments with atherosclerosis, irrespective of stenosis severity (range, 0-11).^33^ Quantitative CAC score was defined per the Agatston method^34,35^ and further categorized as 0, 1 to 99, or 100 or more Agatston units.

#### Carotid Atherosclerosis

Carotid plaque was defined following the Mannheim consensus^36^ and classified as no plaque, unilateral plaque(s), or bilateral plaques.^37^ For spline analysis, a carotid plaque score was calculated as: no plaque = 0, unilateral plaque(s) = 1, and bilateral plaques = 2.^38^

### Covariates

Covariates were identified using a directed acyclic graph (eFigure 2 in Supplement 1).^39^ At conscription, data on age (years), site (n = 6), year of conscription (n = 5 four-year periods), body mass index (BMI, calculated as weight in kilograms divided by height in meters squared), cardiorespiratory fitness (watts), and muscular strength (newtons) were obtained from the Swedish Military Conscription Register.

For individuals reporting a history of smoking in SCAPIS, the duration of smoking at conscription (in years) was calculated based on self-reported age of smoking initiation. Educational attainment at conscription was proxied by self-reported educational level in SCAPIS, classified into the following 4 categories: unfinished primary school, primary school, secondary school, and university degree. Additionally, in SCAPIS, age (years) and site (n = 6) were included as covariates.

### Statistical Analysis

Multinomial logistic regression models were used to estimate odds ratios (ORs) for the association between adolescent BP and atherosclerosis in middle age, with the following 2 levels of adjustment: (1) basic, adjusting for age, site, and year at conscription, as well as age and site in SCAPIS; and (2) extended, meaning the basic model plus BMI and smoking duration at conscription and educational level at SCAPIS. Adolescent BP was modeled both as categorical and continuous variables using restricted cubic splines with knots placed at the fifth, 35th, 65th, and 95th percentiles.^40,41^ To enhance clinical interpretation, as a measure of absolute risk, we estimated the adjusted marginal probability (obtained by marginalization or parametric g-formula) of atherosclerosis across BP categories, hereafter referred as *adjusted prevalence*.^42^

All statistical tests were 2-sided, with *P* < .05 considered statistically significant. Analyses were performed using Stata version 18 (StataCorp).

#### Sensitivity Analyses

To evaluate the robustness of our findings, we conducted several sensitivity analyses (eMethods in Supplement 1).

## Results

A total of 10 222 men (mean [SD] age at conscription, 18.3 [0.5] years) were included in the analyses (Table 1). At conscription, mean (SD) SBP and DBP values were 127.6 (10.7) mm Hg and 68.3 (9.5) mm Hg, respectively, with 1723 participants (16.9%) classified as having stage 2 hypertension. At follow-up in SCAPIS, after a median (IQR) period of 39.5 (35.2-42.8) years’ follow-up, median (IQR) SBP and mean (SD) DBP were 128.0 (118.0-138.0) mm Hg and 78.7 (10.2) mm Hg, respectively, and 2523 participants (24.9%) had a hypertension diagnosis. In middle age, 4943 participants (54.3%) had any coronary stenosis, with 4159 (45.7%) of participants having 1% to 49% coronary stenosis and 784 (8.6%) having 50% or greater coronary stenosis; 4643 (52.0%) had a CAC score greater than 0, and 6180 (60.6%) had carotid plaque(s). Compared to excluded participants, those included had more favorable profiles in terms of smoking status, education, and atherosclerosis burden (eTable 1 in Supplement 1).

**Table 1.** Characteristics of Participants by 2025 American College of Cardiology/American Heart Association Blood Pressure (BP) Categories

Characteristic	No. (%)				
	Total	BP		Hypertension	
		Normal, SBP <120 mm Hg and DBP <80 mm Hg	Elevated, SBP = 120-129 mm Hg and DBP <80 mm Hg	Stage 1, SBP = 130-139 mm Hg or DBP = 80-89 mm Hg	Stage 2, SBP ≥140 mm Hg or DBP ≥90 mm Hg
Participants	10 222 (100)	1898 (18.6)	3088 (30.2)	3513 (34.4)	1723 (16.9)
Baseline (at conscription)					
Age, mean (SD), y	18.3 (0.5)	18.3 (0.5)	18.3 (0.5)	18.4 (0.5)	18.4 (0.5)
Height, mean (SD), cm	179.6 (6.4)	178.8 (6.3)	179.4 (6.3)	179.8 (6.5)	180.6 (6.6)
Weight, mean (SD), kg	68.6 (9.0)	65.9 (7.9)	68.1 (8.5)	69.2 (9.2)	71.2 (9.7)
BMI, mean (SD)^a^	21.2 (2.4)	20.6 (2.1)	21.1 (2.3)	21.4 (2.5)	21.8 (2.6)
BMI categories (n = 10 222)					
Underweight	980 (9.6)	279 (14.7)	278 (9.0)	303 (8.6)	120 (7.0)
Normal weight	8573 (83.9)	1564 (82.4)	2644 (85.6)	2938 (83.6)	1427 (82.8)
Overweight	614 (6.0)	52 (2.7)	156 (5.1)	249 (7.1)	157 (9.1)
Obesity	55 (0.5)	3 (0.2)	10 (0.3)	23 (0.7)	19 (1.1)
Smoking duration, median (IQR), y	0.0 (0.0-1.1)	0.0 (0.0-2.3)	0.0 (0.0-1.4)	0.0 (0.0-0.9)	0.0 (0.0-0.0)
SBP, mean (SD), mm Hg	127.6 (10.7)	113.1 (4.1)	123.4 (2.8)	131.3 (5.4)	143.8 (6.1)
DBP, mean (SD), mm Hg	68.3 (9.5)	64.7 (7.2)	66.0 (7.7)	70.9 (9.9)	71.3 (11.1)
MAP, mean (SD), mm Hg	88.1 (7.7)	80.8 (5.1)	85.1 (5.2)	91.0 (6.2)	95.4 (7.3)
PP, mean (SD), mm Hg	59.3 (13.2)	48.5 (7.9)	57.3 (8.2)	60.3 (12.9)	72.5 (13.8)
Follow-up (at SCAPIS)					
Follow-up, median (IQR), y	39.5 (35.2-42.8)	39.8 (35.2-43.0)	38.7 (35.0-42.3)	39.9 (35.4-43.0)	39.5 (35.4-42.7)
Age, median (IQR), y	57.8 (53.4-61.2)	58.1 (53.4-61.3)	56.8 (53.0-60.7)	58.2 (53.6-61.5)	57.7 (53.5-61.2)
BMI, median (IQR)^a^	26.8 (24.7-29.4)	26.6 (24.5-29.0)	26.8 (24.7-29.4)	26.9 (24.8-29.6)	26.9 (24.8-29.6)
BMI categories (n = 10 222)					
Underweight	9 (0.1)	3 (0.2)	2 (0.1)	3 (0.1)	1 (0.1)
Normal weight	2833 (27.7)	576 (30.4)	846 (27.4)	941 (26.8)	470 (27.3)
Overweight	5202 (50.9)	974 (51.3)	1592 (51.6)	1782 (50.7)	854 (49.6)
Obesity	2178 (21.3)	345 (18.2)	648 (21.0)	787 (22.4)	398 (23.1)
SBP, median (IQR), mm Hg	128.0 (118.0-138.0)	122.0 (114.0-134.0)	126.0 (117.0-136.0)	129.0 (120.0-140.0)	132.0 (122.0-142.0)
DBP, mean (SD), mm Hg	78.7 (10.2)	77.0 (10.5)	78.1 (10.0)	79.5 (10.2)	80.2 (10.0)
Hypertension (n = 10 119)^b^					
Yes	2523 (24.9)	330 (17.5)	668 (21.9)	936 (26.9)	589 (34.5)
No	7596 (75.1)	1553 (82.5)	2380 (78.1)	2543 (73.1)	1120 (65.5)
Antihypertensive medication (n = 10 222)^b^					
Yes	2749 (26.9)	387 (20.4)	750 (24.3)	1004 (28.6)	608 (35.3)
No	7473 (73.1)	1511 (79.6)	2338 (75.7)	2509 (71.4)	1115 (64.7)
Dyslipidemia (n = 10 119)^b^					
Yes	1405 (13.9)	217 (11.5)	392 (12.9)	515 (14.8)	281 (16.4)
No	8714 (86.1)	1666 (88.5)	2656 (87.1)	2964 (85.2)	1428 (83.6)
Hypolipidemic medication (n = 10 222)^b^					
Yes	1408 (13.8)	193 (10.2)	411 (13.3)	520 (14.8)	284 (16.5)
No	8814 (86.2)	1705 (89.8)	2677 (86.7)	2993 (85.2)	1439 (83.5)
Diabetes (n = 10 214)^b^					
Yes	864 (8.5)	129 (6.8)	253 (8.2)	315 (9.0)	167 (9.7)
No	9350 (91.5)	1769 (93.2)	2832 (91.8)	3193 (91.0)	1556 (90.3)
Antidiabetic medication (n = 10 222)^b^					
Yes	470 (4.6)	69 (3.6)	149 (4.8)	168 (4.8)	84 (4.9)
No	9752 (95.4)	1829 (96.4)	2939 (95.2)	3345 (95.2)	1639 (95.1)
Educational level (n = 10 222)					
Unfinished primary school	39 (0.4)	9 (0.5)	11 (0.4)	12 (0.3)	7 (0.4)
Primary school	940 (9.2)	182 (9.6)	266 (8.6)	333 (9.5)	159 (9.2)
Secondary school	5088 (49.8)	968 (51.0)	1565 (50.7)	1723 (49.1)	832 (48.3)
University degree	4155 (40.7)	739 (38.9)	1246 (40.4)	1445 (41.1)	725 (42.1)
Coronary stenosis (n = 9110)					
0%	4167 (45.7)	833 (48.8)	1330 (48.4)	1413 (45.1)	591 (38.8)
1%-49%	4159 (45.7)	761 (44.6)	1193 (43.5)	1432 (45.7)	773 (50.7)
≥50%	784 (8.6)	113 (6.6)	223 (8.1)	287 (9.2)	161 (10.6)
CAC (n = 8925), Agatston units					
0	4282 (48.0)	860 (51.2)	1354 (50.3)	1456 (47.6)	612 (41.0)
1-99	3080 (34.5)	582 (34.6)	901 (33.4)	1027 (33.6)	570 (38.2)
≥100	1563 (17.5)	238 (14.2)	439 (16.3)	574 (18.8)	312 (20.9)
Carotid plaque (n = 10 205)					
No plaque	4025 (39.4)	794 (41.9)	1289 (41.8)	1322 (37.7)	620 (36.1)
Unilateral plaque(s)	3114 (30.5)	590 (31.1)	903 (29.3)	1099 (31.3)	522 (30.4)
Bilateral plaques	3066 (30.0)	511 (27.0)	890 (28.9)	1087 (31.0)	578 (33.6)

Abbreviations: BMI, body mass index; CAC, coronary artery calcium; DBP, diastolic BP; MAP, mean arterial pressure; PP, pulse pressure; SBP, systolic BP; SCAPIS, Swedish Cardiopulmonary Bioimage Study.

^a^ Calculated as weight in kilograms divided by height in meters squared.

^b^ Medication use for hypertension, dyslipidemia, and diabetes in the year prior to SCAPIS measurements was retrieved from the Swedish Prescribed Drug Register. Diagnoses of hypertension and dyslipidemia were based on self-reported, doctor-diagnosed conditions. Diabetes status in SCAPIS was determined from blood sample analyses and categorized as either diabetes or nondiabetes, with the nondiabetes group including normoglycemia, impaired fasting glucose, and elevated hemoglobin A_1c_.

### BP in Adolescence and Coronary Stenosis in Middle Age

**Figure 1.**
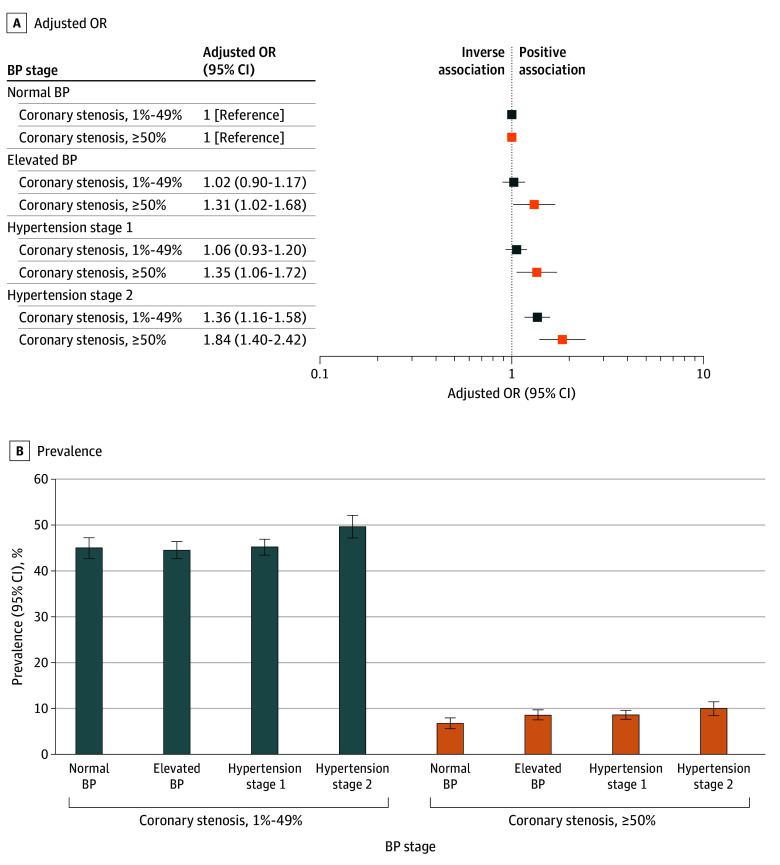
Associations of 2025 American College of Cardiology/American Heart Association (ACC/AHA) Blood Pressure (BP) Categories in Adolescence With Computed Tomography Coronary Stenosis in Middle Age Multinomial logistic models depicting adjusted odds ratios (ORs) (A) and prevalences (B) are adjusted for age at conscription, site in conscription, year of conscription, body mass index at conscription, smoking duration at conscription, age at Swedish Cardiopulmonary Bioimage Study (SCAPIS), site in SCAPIS, and educational level at SCAPIS. Normal BP: SBP <120 mm Hg and DBP <80 mm Hg; elevated BP: SBP = 120-129 mm Hg and DBP <80 mm Hg; hypertension stage 1: SBP = 130-139 mm Hg or DBP = 80-89 mm Hg; and hypertension stage 2: SBP ≥140 mm Hg or DBP ≥90 mm Hg.

After adjustment, a strong dose-response association was observed between 2025 ACC/AHA BP categories and coronary stenosis on CCTA, particularly for severe stenosis (Figure 1, Table 2; eTable 2 in Supplement 1). Adolescents with stage 2 hypertension had an OR of 1.84 (95% CI, 1.40-2.42) and an adjusted prevalence of 10.1% (95% CI, 8.6%-11.5%) for severe stenosis (defined as ≥50% coronary stenosis) compared to 6.9% (95% CI, 5.7%-8.1%) among those with normal BP. Segment-specific analyses across the 11 most relevant coronary arteries showed notable differences in adjusted prevalences, with higher rates in proximal segments (eFigure 3 and eTable 3 in Supplement 1).

**Table 2.** Association of the 2025 American College of Cardiology/American Heart Association (ACC/AHA) Blood Pressure (BP) Categories in Adolescence and Coronary Atherosclerosis in Middle Age

2025 ACC/AHA BP classification	Basic adjustment^a^		Extended adjustment^b^	
	OR (95% CI)	*P* value	OR (95% CI)	*P* value
Coronary stenosis: 1%-49%^c^				
Normal BP^d^	1 [Ref]	NA	1 [Ref]	NA
Elevated BP^d^	1.05 (0.92-1.19)	.48	1.02 (0.90-1.17)	.73
Hypertension stage 1^d^	1.10 (0.97-1.25)	.15	1.06 (0.93-1.20)	.40
Hypertension stage 2^d^	1.43 (1.23-1.66)	<.001	1.36 (1.16-1.58)	<.001
Coronary stenosis: ≥50%^c^				
Normal BP^d^	1 [Ref]	NA	1 [Ref]	NA
Elevated BP^d^	1.35 (1.05-1.73)	.02	1.31 (1.02-1.68)	.03
Hypertension stage 1^d^	1.41 (1.11-1.79)	.01	1.35 (1.06-1.72)	.01
Hypertension stage 2^d^	1.94 (1.48-2.54)	<.001	1.84 (1.40-2.42)	<.001

Abbreviations: NA, not applicable; OR, odds ratio; ref, reference.

^a^ Basic adjusted model: adjusted for age at conscription, age at Swedish Cardiopulmonary Bioimage Study (SCAPIS), site in conscription, site in SCAPIS, and conscription year.

^b^ Extended adjusted model: basic adjustment plus body mass index at conscription (linear and quadratic terms), smoking at conscription, and education level at SCAPIS.

^c^ Reference category for coronary computed tomography angiography stenosis: no stenosis.

^d^ Normal BP: SBP <120 mm Hg and DBP <80 mm Hg; elevated BP: SBP = 120-129 mm Hg and DBP <80 mm Hg; hypertension stage 1: SBP = 130-139 mm Hg or DBP = 80-89 mm Hg; and hypertension stage 2: SBP ≥140 mm Hg or DBP ≥90 mm Hg.

Overall, BP categories showed a dose-response association with all coronary plaque types; stage 2 hypertension had ORs of 1.73 (95% CI, 1.33-2.25), 1.38 (95% CI, 1.18-1.61), and 1.13 (95% CI, 0.72-1.75) for mixed, calcified, and noncalcified plaques, respectively (eFigure 4 in Supplement 1).

Comparable dose-response associations were also seen using 2024 ESC BP categories across all 3 atherosclerotic variables (eFigure 5 in Supplement 1).

### SBP in Adolescence and Coronary Stenosis in Middle Age

**Figure 2.**
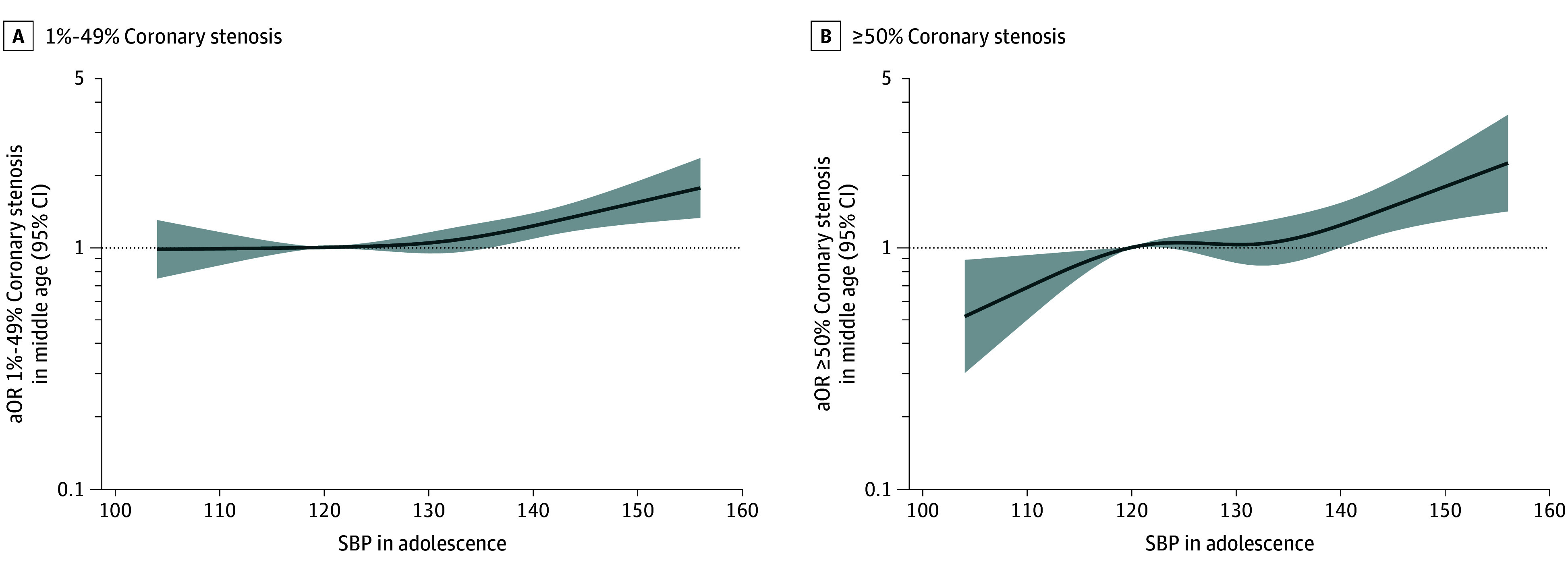
Associations of Restricted Cubic Splines for Systolic Blood Pressure (SBP) in Adolescence With Computed Tomography Coronary Stenosis in Middle Age A, 1%-49% Coronary stenosis; B, ≥50% Coronary stenosis. Multinomial logistic models are adjusted for age at conscription, site in conscription, year of conscription, body mass index at conscription, smoking duration at conscription, age at Swedish Cardiopulmonary Bioimage Study (SCAPIS), site in SCAPIS, and educational level at SCAPIS. X-axes are trimmed to depict the associations for the first to 99th percentile of SBP values. Reference is set at 120 mm Hg. aOR indicates adjusted odds ratio.

Spline models showed a monotonic positive association between adolescent SBP and both coronary and carotid atherosclerosis, with stronger associations for coronary and severe outcomes. Notably, SBP less than 120 mm Hg seemed to be associated with lower risk (Figure 2; eFigure 6 in Supplement 1).

After adjustment, a strong dose-response association was observed between 2025 ACC/AHA SBP categories and CCTA stenosis, particularly for severe cases (eFigure 7 and eTables 4 and 5 in Supplement 1). Adolescents with stage 2 SBP (≥140 mm Hg) had an OR of 1.97 (95% CI, 1.50-2.60) for severe stenosis compared to those with normal SBP (<120 mm Hg). Adjusted prevalences by SBP category across 11 key coronary segments are presented in eTable 6 in Supplement 1.

Comparable dose-response patterns were observed using the 2024 ESC SBP categories across all atherosclerotic variables (eFigure 8 in Supplement 1).

### DBP in Adolescence and Coronary Stenosis in Middle Age

**Figure 3.**
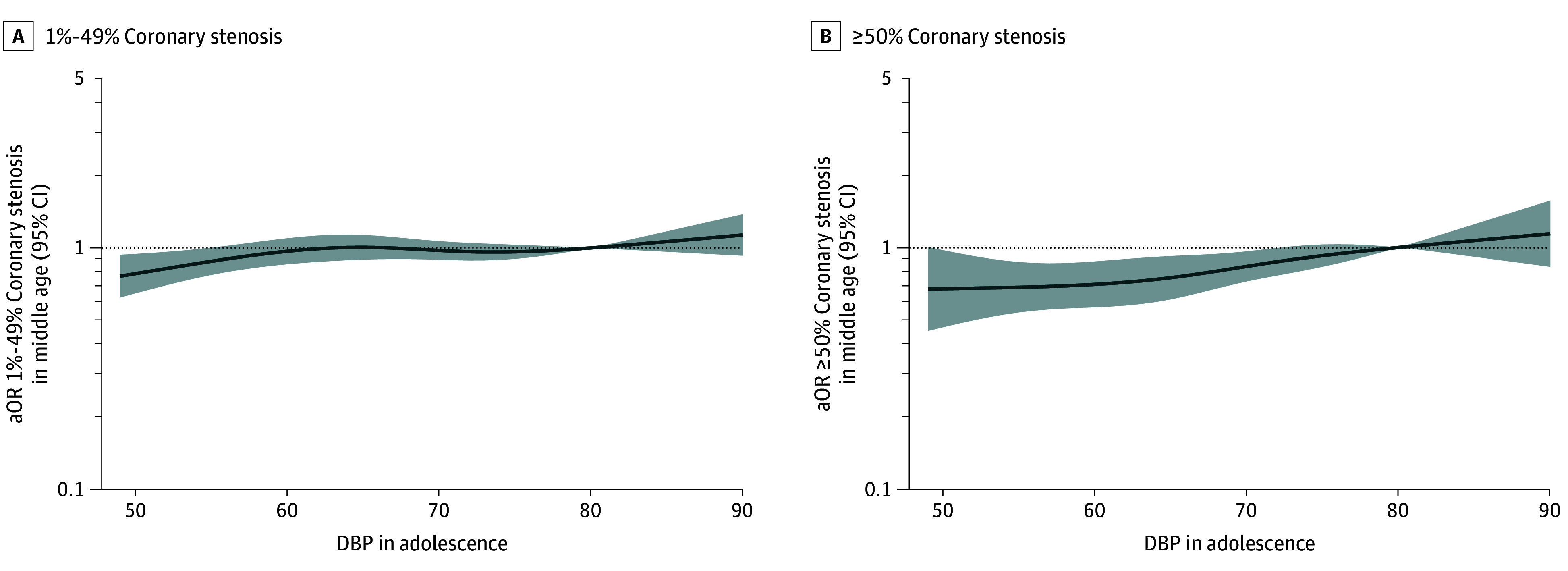
Associations of Restricted Cubic Splines for Diastolic Blood Pressure (DBP) in Adolescence With Computed Tomography Coronary Stenosis in Middle Age A, 1%-49% Coronary stenosis; B, ≥50% Coronary stenosis. Multinomial logistic models are adjusted for age at conscription, site in conscription, year of conscription, body mass index at conscription, smoking duration at conscription, age at Swedish Cardiopulmonary Bioimage Study (SCAPIS), site in SCAPIS, and educational level at SCAPIS. X-axes are trimmed to depict the associations for the first to 99th percentile of DBP values. Reference is set at 80 mm Hg. aOR indicates adjusted odds ratio.

Spline models showed positive associations between adolescent DBP and coronary atherosclerosis, particularly for severe outcomes, but not with carotid plaques, although associations were notably weaker than those observed for SBP. DBP values less than 80 mm Hg were linked to lower odds of severe coronary stenosis and CAC (Figure 3; eFigure 6 in Supplement 1).

After adjustment, the 2025 ACC/AHA DBP categories showed no significant association with severe coronary stenosis, with an OR of 1.06 (95% CI, 0.51-2.19) for stage 2 DBP vs normal DBP (eFigure 9 and eTables 7 and 8 in Supplement 1). Adjusted prevalences across 11 key coronary segments are shown in eTable 9 in Supplement 1.

Comparable but weaker dose-response trends were seen using the 2024 ESC DBP categories (eFigure 10 in Supplement 1).

### Mean Arterial Pressure and Pulse Pressure in Adolescence and Coronary Stenosis in Middle Age

Consistent with SBP, spline models revealed a positive association between mean arterial pressure and atherosclerosis (eFigures 11 and 12 in Supplement 1). In contrast, splines for pulse pressure showed only very weak associations with atherosclerosis indicators (eFigures 12 and 13 in Supplement 1).

### BP in Adolescence and Coronary Artery Calcium and Carotid Plaque in Middle Age

See the eResults in Supplement 1.

### Sensitivity Analyses

Binomial logistic associations of 2025 ACC/AHA BP categories with additional atherosclerosis outcomes (any coronary stenosis, any significant coronary stenosis [≥50%], any noncalcified plaque, segment involvement score ≥4,^33^ and CAC score ≥100) are presented in eTable 10 in Supplement 1, showing comparable associations with multinomial analyses.

Overall, sensitivity analyses considering varying definitions of coronary stenosis yielded similar conclusions, although considering participants with data in all the 11 relevant coronary segments moderately attenuated the associations for 50% or greater coronary stenosis (eTable 11 in Supplement 1). Likewise, sensitivity analyses considering additional levels of adjustment produced comparable findings, although not adjusting for BMI at conscription moderately strengthened the association. In contrast, exclusions of participants with CVD slightly attenuated the association (eTable 12 in Supplement 1). The associations were similar when restricting analyses to participants not taking antihypertensive medication at the measurement in SCAPIS, but associations were fully attenuated toward the null among those receiving treatment (eTable 13 in Supplement 1).

## Discussion

This novel population-based cohort study found strong associations between higher 2025 ACC/AHA and 2024 ESC BP categories in adolescence and greater levels of atherosclerosis nearly 40 years later, even after adjusting for confounders. The association was particularly notable for severe coronary atherosclerosis outcomes, with an increased risk already evident in the elevated BP categories according to the 2025 ACC/AHA and the 2024 ESC BP guidelines. Moreover, the association between SBP and atherosclerosis followed a positive dose-response pattern, which was more pronounced than the associations observed for DBP, mean arterial pressure, or pulse pressure.

This is, to our knowledge, the first study to examine the association between adolescent BP and CCTA-determined atherosclerosis later in life. Adolescents with stage 2 hypertension (≥140/90 mm Hg) had ORs of 1.36 and 1.84 for 1% to 49% and 50% or greater coronary stenosis in middle age, respectively. Among adolescents with normal BP, 45.0% developed 1% to 49% stenosis and 6.9% developed 50% or greater stenosis, compared to 49.7% and 10.1% in the stage 2 hypertension group, corresponding to absolute differences of 4.7% and 3.2%. For context, while considering the differences in the time frame, the SPRINT trial,^43^ a landmark randomized clinical trial comparing intensive vs standard BP targets in high-risk hypertensive adults, considered a mere 1.6% absolute difference in event rates clinically significant. Moreover, even small absolute differences when applied to large populations could translate into substantial public health benefits.

In 2017, the ACC/AHA guidelines lowered the threshold to define hypertension to 130/80 or greater mm Hg and defined normal BP as less than 120/80 mm Hg.^24^ In 2024, the ESC guidelines aligned more closely with the 2017 ACC/AHA classification, now defining normal BP as less than 120/70 mm Hg.^26^ Our study supports the clinical significance of the American and European elevated BP categories (SBP ≥120 mm Hg), demonstrating that even modest elevations in BP are associated with an increased risk for atherosclerosis based on CCTA and should not be regarded as normal. This aligns with our restricted cubic spline analysis, which showed a fairly linear positive association between SBP and DBP and atherosclerosis, with lower BP consistently associated with less atherosclerosis, particularly severe coronary disease. Similarly, the newly released 2025 ACC/AHA guidelines reaffirm the 2017 thresholds, emphasizing that BP less than 120/80 mm Hg remains the optimal target and supporting early intervention even for modest BP elevations.^27^

In our study, the association between SBP and atherosclerosis was stronger than that observed for DBP, mean arterial pressure, or pulse pressure. The relative importance of SBP vs DBP for CVD risk remains debated, particularly whether isolated systolic hypertension in adolescence is a true risk factor or an artifact of pulse wave reflections in elastic arteries.^44,45^ Several studies have shown a stronger association between SBP in adolescence and CAC later in life compared to DBP.^19,46^ Moreover, the greater contribution of SBP was also found for the association with CVD events,^11^ although a large study also analyzing Swedish conscripts while accounting for familial confounders found similar associations for SBP and DBP.^47^ The previously mentioned higher contribution of SBP contrasts with 2 large studies also analyzing Swedish military conscripts, which found that DBP was more closely associated with CVD events and CVD mortality.^13,14^ However, while these studies relied on registry-based outcomes (thus more prone to diagnostic bias), SCAPIS provides imaging-based data specifically designed to assess subclinical atherosclerosis. Despite these discrepancies, the accumulating evidence linking elevated SBP in adolescence to subclinical atherosclerosis and future CVD risk underscores the importance of considering SBP in adolescence as a critical factor in early cardiovascular risk assessment.

To our knowledge, no previous study has examined the association between BP in adolescence and CCTA stenosis in middle age. However, several studies have investigated its association with CAC. The Cardiovascular Risk in Young Finns Study,^19^ which followed up 589 individuals for 27 years, found that SBP in adolescence was associated with the presence of CAC in adulthood independently of subsequent changes in cardiovascular risk factors, with an OR of 1.38 per 1-SD increase in adolescent SBP. The Muscatine Study also found a positive association between SBP in childhood and young adulthood and CAC in young adulthood.^48^ Beyond CAC, a meta-analysis reported similar findings for carotid intima-media thickness, pulse wave velocity, and left ventricular hypertrophy.^49^ Notably, the association between adolescent BP extended not only to atherosclerotic CVD,^10,47^ but also to nonatherosclerotic outcomes, including heart failure,^13,47^ chronic kidney disease,^50^ and dementia,^51^ as well as to CVD mortality.^8,14,52^

Our study builds upon previous evidence by analyzing a large, randomly selected cohort of more than 9000 individuals, evaluating stenosis in the 11 most relevant coronary segments using the noninvasive CCTA. This is important, as different coronary segments carry distinct risks for myocardial infarction, refining our understanding of the impact of adolescent BP on atherosclerosis.^21^ In addition, CCTA enables the characterization of not only calcified atherosclerosis, but also noncalcified and mixed atherosclerosis, 2 phenotypes that appear more prone to rupture and to initiate cardiovascular events.^20,21^ While the relatively low prevalence of noncalcified plaques (2.7% of the total population) limits direct comparison, our findings suggest that adolescent BP is strongly associated with both calcified and (particularly) mixed composition. This raises the hypothesis that BP may be especially relevant for the development of mixed plaque phenotypes. Further studies using high-resolution atherosclerotic imaging are warranted to better characterize the phenotype at a coronary segment level, rather than across the entire coronary tree. Studies examining longitudinal BP data would also be of interest, as they could complement the role of adolescent BP by clarifying how changes from adolescence into adulthood influence atherosclerosis progression and the risk of subsequent cardiovascular events. Altogether, our study strengthens the biological plausibility of the detrimental effects of BP in early life, linking adolescent BP elevation with adult CVD. By examining anatomical intermediate end points, such as CCTA-defined atherosclerosis, our findings provide mechanistic insight into CVD development, identify opportunities for earlier prevention, and support the evolving focus on atherosclerosis-driven risk assessment rather than solely functional ischemia evaluation.^22,23^

### Strength and Limitations

The primary strength of this study lies in the use of CCTA on a population-wide scale within a large, representative sample. Furthermore, the protocol does not rely on electronic health records or registry data but instead assesses CCTA in all participants, thereby limiting health-seeking bias. The nearly 40-year follow-up of a young cohort also reduces the risk of reverse causation, as it is unlikely that adolescent atherosclerosis would lead to elevated BP.

Several limitations should be noted. First, as conscription was mandatory only for men before 2010, the study included only male participants. In SCAPIS,^32^ atherosclerosis was substantially more prevalent in men than in women, with any coronary stenosis observed in 55.1% of men vs 29.3% of women and severe stenosis (≥50%) in 8.3% vs 2.2%, respectively. Given these sex differences in atherosclerosis burden, distinct BP trajectories across the life course,^53^ and the possibility of sex-BP interactions,^17,53^ future studies in women are needed to more appropriately evaluate the associations between BP in adolescence and atherosclerosis. Second, BP was measured during conscription, a potentially stressful context, which may have introduced a white coat effect.^54^ This could increase BP variability and possibly attenuate the observed associations. Third, the small number of participants with elevated DBP precludes the categorization of BP into separate systolic and diastolic phenotypes. Fourth, although we used CCTA, plaque characterization was limited. Mixed plaque composition was assessed only at the coronary tree level, not at individual segments, and carotid plaque data lacked detail on phenotype, number, or stenosis severity. Finally, residual confounding remains possible, particularly from unmeasured variables at conscription, such as blood lipids and glucose levels, alcohol consumption, physical activity, diet, or antihypertensive medication. Given that few adolescents were expected to use such medication in the 1980s or 1990s, any resulting bias is likely to be small. Additionally, an analysis restricted to SCAPIS participants on antihypertensive medication attenuated the association between adolescent BP and coronary stenosis, consistent with a suppressive mediation—higher early-life BP increases the likelihood of treatment, which in turn may reduce atherosclerosis development. Overall, these observations suggest that our estimates may, if anything, underestimate the true associations.

## Conclusions

In conclusion, this population-based cohort study found that higher BP in adolescence was associated with an increased risk of coronary atherosclerosis in late adulthood, particularly for SBP in relation to severe coronary stenosis. Thus, elevated BP already in adolescence, as defined by current clinical guidelines, is strongly linked to a higher risk for atherosclerosis in middle age.

## Notes

Supplement 1. eMethods. Supplementary Methods eResults. Supplementary Results eTable 1. Characteristics of Included vs Excluded Participants eTable 2. Multinomial Logistic Regression, Adjusted Prevalences for the Association of 2025 ACC/AHA Blood Pressure Categories in Adolescence With Coronary and Carotid Atherosclerosis in Middle Age eTable 3. Multinomial Logistic Models, Adjusted Prevalences for the Association of 2025 ACC/AHA Blood Pressure Categories in Adolescence With Coronary Stenosis in Middle Age in the 11 Most Relevant Coronary Segments eTable 4. Multinomial Logistic Regression, Odds Ratios for the Association of 2025 ACC/AHA Systolic Blood Pressure Categories in Adolescence With Coronary and Carotid Atherosclerosis in Middle Age eTable 5. Multinomial Logistic Regression, Adjusted Prevalences for the Association of 2025 ACC/AHA Systolic Blood Pressure Categories in Adolescence With Coronary and Carotid Atherosclerosis in Middle Age eTable 6. Multinomial Logistic Models, Adjusted Prevalences for the Association of 2025 ACC/AHA Systolic Blood Pressure Categories in Adolescence With Coronary Stenosis in Middle Age in the 11 Most Relevant Coronary Segments eTable 7. Multinomial Logistic Regression, Odds Ratios for the Association of 2025 ACC/AHA Diastolic Blood Pressure Categories in Adolescence With Coronary and Carotid Atherosclerosis in Middle Age eTable 8. Multinomial Logistic Regression, Adjusted Prevalences for the Association of 2025 ACC/AHA Diastolic Blood Pressure Categories in Adolescence With Coronary and Carotid Atherosclerosis in Middle Age eTable 9. Multinomial Logistic Models, Adjusted Prevalences for the Association of 2025 ACC/AHA Diastolic Blood Pressure Categories in Adolescence With Coronary Stenosis in Middle Age in the 11 Most Relevant Coronary Segments eTable 10. Associations of 2025 ACC/AHA Blood Pressure Categories in Adolescence With Coronary Atherosclerosis in Middle Age Considering Binomial Atherosclerosis Categories eTable 11. Sensitivity Analysis, Associations of 2025 ACC/AHA Blood Pressure Categories in Adolescence With Coronary Stenosis in Middle Age Considering Influential Factors for Defining Coronary Stenosis eTable 12. Sensitivity Analysis, Associations of 2025 ACC/AHA Blood Pressure Categories in Adolescence With Coronary Stenosis in Middle Age Considering Additional Levels of Adjustment and Excluding Participants With Cardiovascular Disease eTable 13. Sensitivity Analysis, Associations of 2025 ACC/AHA Blood Pressure Categories in Adolescence With Coronary Stenosis in Middle Age Considering Antihypertensive Medication in Middle Age eTable 14. Multinomial Logistic Regression, Odds Ratios for the Association of 2025 ACC/AHA Blood Pressure Categories in Adolescence With Coronary Artery Calcium and Carotid Atherosclerosis in Middle Age eFigure 1. Flowchart of the Study eFigure 2. Directed Acyclic Graph for the Association of Blood Pressure in Adolescence With Atherosclerosis in Middle Age eFigure 3. Adjusted Prevalences for the Association of 2025 ACC/AHA Blood Pressure Categories in Adolescence With Coronary Stenosis in Middle Age in the 11 Most Relevant Coronary Segments eFigure 4. Associations of 2025 ACC/AHA Blood Pressure Categories in Adolescence With Composition of the Coronary Plaques in Middle Age eFigure 5. Associations of 2024 ESC Blood Pressure Categories in Adolescence With Coronary and Carotid Atherosclerosis in Middle Age eFigure 6. Associations Across Restricted Cubic Splines of Systolic and Diastolic Blood Pressures in Adolescence With Coronary (Segment Involvement Score and CAC Score) and Carotid (Carotid Plaque Score) Atherosclerosis in Middle Age eFigure 7. Associations of 2025 ACC/AHA Systolic Blood Pressure Categories in Adolescence With Coronary and Carotid Atherosclerosis in Middle Age eFigure 8. Associations of 2024 ESC Systolic Blood Pressure Categories in Adolescence With Coronary and Carotid Atherosclerosis in Middle Age eFigure 9. Associations of 2025 ACC/AHA Diastolic Blood Pressure Categories in Adolescence With Coronary and Carotid Atherosclerosis in Middle Age eFigure 10. Associations of 2024 ESC Diastolic Blood Pressure Categories in Adolescence With Coronary and Carotid Atherosclerosis in Middle Age eFigure 11. Associations Across Restricted Cubic Splines of Mean Arterial Pressure in Adolescence With Coronary and Carotid Atherosclerosis in Middle Age eFigure 12. Associations Across Restricted Cubic Splines of Mean Arterial Pressure and Pulse Pressure in Adolescence With Coronary (Segment Involvement Score and CAC Score) and Carotid (Carotid Plaque Score) Atherosclerosis in Middle Age eFigure 13. Associations Across Restricted Cubic Splines of Pulse Pressure in Adolescence With Coronary and Carotid Atherosclerosis in Middle Age eFigure 14. Associations of 2025 ACC/AHA Blood Pressure Categories in Adolescence With CAC Score and Carotid Plaque in Middle Age eFigure 15. Associations Across Restricted Cubic Splines of Systolic Blood Pressure in Adolescence With CAC Score and Carotid Plaques in Middle Age eFigure 16. Associations Across Restricted Cubic Splines of Diastolic Blood Pressure in Adolescence With CAC Score and Carotid Plaques in Middle Age eReferences.

Supplement 2. Data Sharing Statement
