# Supplementary material for: Blood Pressure in Adolescence and Atherosclerosis in Middle Age
Source: JAMA Cardiol. 2025 Nov 19;11(1):14–24. doi: 10.1001/jamacardio.2025.4271 (PMC12631567; doi:10.1001/jamacardio.2025.4271)
Supplement: Supplement 1. — eMethods. Supplementary Methods eResults. Supplementary Results eTable 1. Characteristics of Included vs Excluded Participants eTable 2. Multinomial Logistic Regression, Adjusted Prevalences for the Association of 2025 ACC/AHA Blood Pressure Categories in Adolescence With Coronary and Carotid Atherosclerosis in Middle Age eTable 3. Multinomial Logistic Models, Adjusted Prevalences for the Association of 2025 ACC/AHA Blood Pressure Categories in Adolescence With Coronary Stenosis in Middle Age in the 11 Most Relevant Coronary Segments eTable 4. Multinomial Logistic Regression, Odds Ratios for the Association of 2025 ACC/AHA Systolic Blood Pressure Categories in Adolescence With Coronary and Carotid Atherosclerosis in Middle Age eTable 5. Multinomial Logistic Regression, Adjusted Prevalences for the Association of 2025 ACC/AHA Systolic Blood Pressure Categories in Adolescence With Coronary and Carotid Atherosclerosis in Middle Age eTable 6. Multinomial Logistic Models, Adjusted Prevalences for the Association of 2025 ACC/AHA Systolic Blood Pressure Categories in Adolescence With Coronary Stenosis in Middle Age in the 11 Most Relevant Coronary Segments eTable 7. Multinomial Logistic Regression, Odds Ratios for the Association of 2025 ACC/AHA Diastolic Blood Pressure Categories in Adolescence With Coronary and Carotid Atherosclerosis in Middle Age eTable 8. Multinomial Logistic Regression, Adjusted Prevalences for the Association of 2025 ACC/AHA Diastolic Blood Pressure Categories in Adolescence With Coronary and Carotid Atherosclerosis in Middle Age eTable 9. Multinomial Logistic Models, Adjusted Prevalences for the Association of 2025 ACC/AHA Diastolic Blood Pressure Categories in Adolescence With Coronary Stenosis in Middle Age in the 11 Most Relevant Coronary Segments eTable 10. Associations of 2025 ACC/AHA Blood Pressure Categories in Adolescence With Coronary Atherosclerosis in Middle Age Considering Binomial Atherosclerosis Categories eTable 11. Sensitivity Analy [file jamacardiol-e254271-s001.pdf]

Supplemental Online Content

Herraiz-Adillo Á, Eriksson H, Ahlqvist VH, et al. Blood pressure in adolescence and atherosclerosis in middle age. *JAMA Cardiol*. Published online November 19, 2025. doi:10.1001/jamacardio.2025.4271

eMethods. Supplementary Methods
eResults. Supplementary Results
eTable 1. Characteristics of Included vs Excluded Participants
eTable 2. Multinomial Logistic Regression, Adjusted Prevalences for the Association of 2025 ACC/AHA Blood Pressure Categories in Adolescence With Coronary and Carotid Atherosclerosis in Middle Age
eTable 3. Multinomial Logistic Models, Adjusted Prevalences for the Association of 2025 ACC/AHA Blood Pressure Categories in Adolescence With Coronary Stenosis in Middle Age in the 11 Most Relevant Coronary Segments
eTable 4. Multinomial Logistic Regression, Odds Ratios for the Association of 2025 ACC/AHA Systolic Blood Pressure Categories in Adolescence With Coronary and Carotid Atherosclerosis in Middle Age
eTable 5. Multinomial Logistic Regression, Adjusted Prevalences for the Association of 2025 ACC/AHA Systolic Blood Pressure Categories in Adolescence With Coronary and Carotid Atherosclerosis in Middle Age
eTable 6. Multinomial Logistic Models, Adjusted Prevalences for the Association of 2025 ACC/AHA Systolic Blood Pressure Categories in Adolescence With Coronary Stenosis in Middle Age in the 11 Most Relevant Coronary Segments
eTable 7. Multinomial Logistic Regression, Odds Ratios for the Association of 2025 ACC/AHA Diastolic Blood Pressure Categories in Adolescence With Coronary and Carotid Atherosclerosis in Middle Age
eTable 8. Multinomial Logistic Regression, Adjusted Prevalences for the Association of 2025 ACC/AHA Diastolic Blood Pressure Categories in Adolescence With Coronary and Carotid Atherosclerosis in Middle Age

© 2025 Herraiz-Adillo Á et al. *JAMA Cardiology*.

eTable 9. Multinomial Logistic Models, Adjusted Prevalences for the Association of 2025 ACC/AHA Diastolic Blood Pressure Categories in Adolescence With Coronary Stenosis in Middle Age in the 11 Most Relevant Coronary Segments

eTable 10. Associations of 2025 ACC/AHA Blood Pressure Categories in Adolescence With Coronary Atherosclerosis in Middle Age Considering Binomial Atherosclerosis Categories

eTable 11. Sensitivity Analysis, Associations of 2025 ACC/AHA Blood Pressure Categories in Adolescence With Coronary Stenosis in Middle Age Considering Influential Factors for Defining Coronary Stenosis

eTable 12. Sensitivity Analysis, Associations of 2025 ACC/AHA Blood Pressure Categories in Adolescence With Coronary Stenosis in Middle Age Considering Additional Levels of Adjustment and Excluding Participants With Cardiovascular Disease

eTable 13. Sensitivity Analysis, Associations of 2025 ACC/AHA Blood Pressure Categories in Adolescence With Coronary Stenosis in Middle Age Considering Antihypertensive Medication in Middle Age

eTable 14. Multinomial Logistic Regression, Odds Ratios for the Association of 2025 ACC/AHA Blood Pressure Categories in Adolescence With Coronary Artery Calcium and Carotid Atherosclerosis in Middle Age

eFigure 1. Flowchart of the Study

eFigure 2. Directed Acyclic Graph for the Association of Blood Pressure in Adolescence With Atherosclerosis in Middle Age

eFigure 3. Adjusted Prevalences for the Association of 2025 ACC/AHA Blood Pressure Categories in Adolescence With Coronary Stenosis in Middle Age in the 11 Most Relevant Coronary Segments

eFigure 4. Associations of 2025 ACC/AHA Blood Pressure Categories in Adolescence With Composition of the Coronary Plaques in Middle Age

eFigure 5. Associations of 2024 ESC Blood Pressure Categories in Adolescence With Coronary and Carotid Atherosclerosis in Middle Age

eFigure 6. Associations Across Restricted Cubic Splines of Systolic and Diastolic Blood Pressures in Adolescence With Coronary (Segment Involvement Score and CAC Score) and Carotid (Carotid Plaque Score) Atherosclerosis in Middle Age

eFigure 7. Associations of 2025 ACC/AHA Systolic Blood Pressure Categories in Adolescence With Coronary and Carotid Atherosclerosis in Middle Age

© 2025 Herraiz-Adillo Á et al. *JAMA Cardiology*.

eFigure 8. Associations of 2024 ESC Systolic Blood Pressure Categories in Adolescence With Coronary and Carotid Atherosclerosis in Middle Age
eFigure 9. Associations of 2025 ACC/AHA Diastolic Blood Pressure Categories in Adolescence With Coronary and Carotid Atherosclerosis in Middle Age
eFigure 10. Associations of 2024 ESC Diastolic Blood Pressure Categories in Adolescence With Coronary and Carotid Atherosclerosis in Middle Age
eFigure 11. Associations Across Restricted Cubic Splines of Mean Arterial Pressure in Adolescence With Coronary and Carotid Atherosclerosis in Middle Age
eFigure 12. Associations Across Restricted Cubic Splines of Mean Arterial Pressure and Pulse Pressure in Adolescence With Coronary (Segment Involvement Score and CAC Score) and Carotid (Carotid Plaque Score) Atherosclerosis in Middle Age
eFigure 13. Associations Across Restricted Cubic Splines of Pulse Pressure in Adolescence With Coronary and Carotid Atherosclerosis in Middle Age
eFigure 14. Associations of 2025 ACC/AHA Blood Pressure Categories in Adolescence With CAC Score and Carotid Plaque in Middle Age
eFigure 15. Associations Across Restricted Cubic Splines of Systolic Blood Pressure in Adolescence With CAC Score and Carotid Plaques in Middle Age
eFigure 16. Associations Across Restricted Cubic Splines of Diastolic Blood Pressure in Adolescence With CAC Score and Carotid Plaques in Middle Age
eReferences.

This supplemental material has been provided by the authors to give readers additional information about their work.

© 2025 Herraiz-Adillo Á et al. *JAMA Cardiology*.

eMethods. Supplementary Methods

Measurement and categorization of blood pressure in adolescence

Blood pressure (BP) at conscription was measured following a standardized protocol.[1] Participants rested in a supine position for 5 to 10 minutes before measurement (coefficients of variation for systolic and diastolic BP were 8.4% and 13.9%, respectively). An appropriately sized cuff was placed at heart level, and trained nurses or physicians performed BP assessments using auscultation to detect Korotkoff sounds.

If systolic BP (SBP) was ≤145 mmHg and diastolic BP (DBP) was between 50 and 85 mmHg, a single measurement was recorded. For values outside these ranges, a second measurement was taken, with the latter used for analysis. BP values were typically rounded to the nearest even number, although rounding to 5 or 10 mmHg occasionally occurred.

BP was categorized according to the 2025 ACC/AHA guidelines[2] into four mutually exclusive categories (without separating systolic and diastolic phenotypes):

- Normal BP: SBP <120 mmHg and DBP <80 mmHg
- Elevated BP: SBP 120–129 mmHg and DBP <80 mmHg
- Hypertension Stage 1: SBP 130–139 mmHg or DBP 80–89 mmHg
- Hypertension Stage 2: SBP ≥140 mmHg or DBP ≥90 mmHg

Additionally, BP was categorized using the 2024 ESC guidelines[3] into three mutually exclusive categories:

- Non-elevated BP: SBP <120 mmHg and DBP <70 mmHg
- Elevated BP: SBP 120–139 mmHg and DBP 70–89 mmHg
- Hypertension: SBP ≥140 mmHg or DBP ≥90 mmHg

Mean arterial pressure was calculated using the formula: MAP = DBP + ((SBP - DBP) / 3), while pulse pressure was calculated as the difference between SBP and DBP.

In line with previous literature, values considered unrealistic were excluded: SBP <80 mmHg (n = 63), DBP <30 mmHg (n = 64),[4] and cases where DBP exceeded SBP (n = 2).

Measurement and categorization of atherosclerosis in middle age

Coronary atherosclerosis

The imaging protocol for SCAPIS has been published elsewhere.[5] Imaging was conducted using a dual-source CT scanner equipped with a Stellar Detector (Somatom Definition Flash, Siemens Medical Solutions).

Contrast-enhanced CCTA was performed at 100 or 120 kV, applying one of five protocols tailored to heart rate, heart rate variability, calcification presence, and body weight. CCTA images were reconstructed using the I26f medium smooth advanced smoothing algorithm and visually assessed for atherosclerosis with the syngo.via software. The degree of luminal obstruction was judged visually as percent diameter coronary stenosis, averaged from the longest and shortest diameter. In SCAPIS, coronary atherosclerosis was characterized following the Society of Cardiovascular Computed Tomography guidelines, which define the coronary vasculature as 18 segments.[6] 1154 participants with technical failures in any of the four proximal segments (1, 5, 6, or 11) on the CCTA images were excluded from the main analysis of coronary plaques and CAC scores.

Participants with calcium blooming artifacts were categorized as 1-49% stenosis, as this artefact usually overestimates stenosis severity. In contrast, participants with coronary stents were classified as ≥50% stenosis, as stents usually indicate severe stenosis. For the primary analysis, CCTA stenosis was defined based on the segment with the highest degree of stenosis among the 11 key coronary segments in the arterial tree (segments 1, 2, 3, 5, 6, 7, 9, 11, 12, 13, and 17). Among the 9110 selected participants, 82.9% had data for all 11 segments, 10.9% had data for 10 segments, 4.2% for 9 segments, and 2.1% had data for ≤8 segments. CCTA stenosis was categorized as no stenosis, 1-49% (less severe) stenosis and ≥50% (severe) stenosis.[7] In addition, for linear splines, a segment involvement score was calculated based on the total number of coronary segments with atherosclerosis, irrespective of stenosis severity (range 0–11).[8] At the level of the arterial tree, coronary atherosclerosis was further classified based on segment plaque composition into the following categories: no detectable plaque, only non-calcified plaque/s (all observed plaques were non-calcified), only calcified plaque/s (all observed plaques were calcified), and mixed composition (a combination of both calcified and non-calcified stenotic segments within the arterial tree).

CAC scoring was performed using non-contrast, electrocardiogram-gated CT imaging at 120 kV. Images were reconstructed with the B35f HeartView medium CaScore algorithm and analyzed using the syngo.via calcium scoring software (Volume Wizard; Siemens) according to the Agatston protocol.[9,10] The Agatston score is calculated by multiplying the area of each calcified lesion (in mm²) by a density factor based on its peak attenuation measured in Hounsfield units (HU), where 130–199 HU = 1, 200–299 HU = 2, 300–399 HU = 3, and ≥400 HU = 4. The quantitative CAC score was further categorized as follows: 0, 1-99 (less severe) or ≥100 (severe) Agatston units.

Carotid atherosclerosis

© 2025 Herraiz-Adillo Á et al. *JAMA Cardiology*.

Carotid artery images were obtained using a standardized protocol with a Siemens Acuson S2000 ultrasound scanner and a 9L4 linear transducer (Siemens, Forchheim, Germany), analyzed by trained operators.[5] Extracranial carotid plaques were assessed via two-dimensional greyscale ultrasound images of the common carotid artery, bulb, and internal carotid artery on both sides. Only subjects with valid readings for both the right and left carotid arteries were included in the analysis.

Carotid plaque was defined, following the Mannheim consensus, as any focal structure protruding into the arterial lumen by at least 0.5 mm or 50% of the surrounding intima-media thickness, or demonstrating a thickness >1.5 mm, measured from the media-adventitia interface to the intima-lumen interface.[11] Participants were classified as having no plaque, unilateral (less severe) plaque/s, or bilateral (severe) plaques.[12] For spline analysis, a carotid plaque score was calculated as follows: no plaque = 0, unilateral plaque/s = 1, and bilateral plaques = 2.[13]

Covariates

Covariates were identified using a directed acyclic graph (DAG) created with DAGitty (https://dagitty.net), see eFigure 2).[13] At conscription, data on age (years), site (n = 6), year of conscription, body mass index (BMI) (kg/m²), cardiorespiratory fitness (watts), and muscular strength (newtons) were obtained from the Swedish Military Conscription Register. The year of conscription, spanning 1969 to 1987, was grouped into five four-year periods to account for temporal trends (1969-1972, 1973-1976, 1977-1980, 1981-1984, 1985-1987). Participants with BMI values ≤14 kg/m² or ≥60 kg/m² were excluded, consistent with previous literature,[13] and linear and quadratic terms in BMI were considered for adjustment. Cardiorespiratory fitness was assessed using a maximal bicycle ergometer test for those participants having a normal resting electrocardiogram. Participants cycled at 60–70 rpm for 5 minutes with resistance adjusted to body weight, followed by incremental increases of 25 watts per minute until volitional exhaustion. The peak workload, expressed in watts, was recorded as the final measure. As an indicator of lean body mass, muscular strength at conscription was evaluated through three different measures: knee extension, handgrip, and elbow flexion strength, performed on an isometric dynamometer at maximal contraction capacity.[4]

For individuals reporting a history of smoking in SCAPIS, the duration of smoking at conscription (years) was calculated based on self-reported age of smoking initiation. Educational attainment at conscription was proxied by self-reported highest level of education in SCAPIS, classified into four categories: unfinished primary school, primary school, secondary school, and university degree. Additionally, at SCAPIS, age (years) and site (Gothenburg, Linköping, Malmö/Lund, Stockholm, Umeå, and Uppsala) were included as covariates.

Sensitivity analyses

In addition to multinomial logistic models, to allow comparability with previous literature,[14] we performed binomial logistic models considering binary atherosclerosis categories: any coronary stenosis (≥1% stenosis), any significant coronary stenosis (≥50% stenosis), any non-calcified plaque, segment involvement score ≥4 coronary segments,[8] and CAC score ≥100 Agatston units.

To evaluate the robustness of our findings, we assessed the impact of varying definitions of coronary stenosis by: 1) including only participants with data from all 11 relevant coronary segments, 2) analysing data from any of the 18 coronary segments, 3) classifying calcium blooming as ≥50% stenosis, and 4) excluding coronary segments with stents. Second, we examined the impact of additional adjustments and exclusions by: 1) omitting adjustment for BMI at conscription, 2) adjusting for cardiorespiratory fitness and muscular strength at conscription,14 3) adjusting for self-reported family history of myocardial infarction and stroke, 4) excluding participants with CVD in SCAPIS, and 5) considering antihypertensive medication at SCAPIS.

© 2025 Herraiz-Adillo Á et al. *JAMA Cardiology*.

eResults. Supplementary Results

Blood pressure in adolescence and coronary artery calcium and carotid plaque in middle age

After extended adjustment, a strong dose–response association was observed between 2025 ACC/AHA BP categories and CAC as well as carotid plaques, particularly for severe atherosclerosis, eTable 14 in Supplement 1. Among adolescents with stage 2 hypertension, the ORs were 1.34 (1.14–1.58) for CAC 1–99 and 1.73 (1.40–2.13) for CAC ≥100 Agatston units (eFigure 14). Associations with carotid plaques were somewhat weaker, with ORs of 1.16 (0.98–1.36) for unilateral plaques and 1.51 (1.27–1.78) for bilateral plaques (eFigure 14). Comparable dose–response relationships were observed when using 2024 ESC BP categories (eFigure 5).

In relation to SBP, spline models showed a monotonic, positive association between adolescent SBP and both CAC and carotid plaques, although associations were attenuated for unilateral carotid plaque/s (eFigure 15). After extended adjustment, strong dose–response associations were observed across 2025 ACC/AHA SBP categories, particularly for severe atherosclerosis: OR 1.78 (1.44–2.20) for severe CAC in those with stage 2 SBP. Associations with carotid plaques were weaker; the OR for bilateral plaques was 1.53 (1.29–1.81) (eFigure 7). Similar dose–response patterns were observed using 2024 ESC BP categories (eFigure 8).

In contrast to SBP, DBP showed attenuated associations in spline models with CAC and carotid plaques (eFigure 16). After extended adjustment, stage 2 DBP was associated with a higher risk of severe CAC, OR 1.80 (1.09–2.97), whereas the association with bilateral carotid plaques was not statistically significant, OR 1.28 (0.83–1.97) (eFigure 9). Similar dose–response relationships were observed using 2024 ESC BP categories (eFigure 10).

© 2025 Herraiz-Adillo Á et al. *JAMA Cardiology*.

eTable 1. Characteristics of Included vs Excluded Participants

	Total	Included		Excluded	
**No. (%)**	10 802 (100.0%)	10 222 (94.6%)		580 (5.4%)	
**Baseline at conscription**					
Age, mean ± SD, years		10 222	18.3 ± 0.5	580	18.4 ± 0.6
Height, mean ± SD, cm		10 222	179.6 ± 6.4	505	179.1 ± 8.1
Weight, mean ± SD, kg		10 222	68.6 ± 9.0	505	68.5 ± 8.7
BMI, mean ± SD, kg/m2		10 222	21.2 ± 2.4	504	21.3 ± 2.4
BMI categories, No. (%)		10 222		504	
Underweight			980 (9.6)		48 (9.5)
Normal weight			8573 (83.9)		423 (83.9)
Overweight			614 (6.0)		29 (5.8)
Obesity			55 (0.5)		4 (0.8)
Smoke duration, median ± IQR, years		10 222	0.0 ± 1.1	191	1.8 ± 5.0
SBP, mean ± SD, mmHg		10 222	127.6 ± 10.7	505	126.5 ± 10.5
DBP, mean ± SD, mmHg		10 222	68.3 ± 9.5	501	68.1 ± 9.7
MAP, mean ± SD, mmHg		10 222	88.1 ± 7.7	501	87.6 ± 7.9
PP, mean ± SD, mmHg		10 222	59.3 ± 13.2	501	58.4 ± 12.8
**Follow-up at SCAPIS**					
Follow-up, median ± IQR, years		10 222	39.5 ± 7.5	580	39.7 ± 7.6
Age, median ± IQR, years		10 222	57.8 ± 7.8	580	58.0 ± 8.1
BMI, median ± IQR, kg/m2		10 222	26.8 ± 4.7	579	26.9 ± 4.9
BMI categories, No. (%)		10 222		79	
Underweight			9 (0.1)		-
Normal weight			2833 (27.7)		23 (29.1)
Overweight			5202 (50.9)		40 (50.6)
Obesity			2178 (21.3)		16 (20.3)
SBP, median ± IQR, mmHg		10 219	128.0 ± 20.0	539	128.0 ± 22.0
DBP, mean ± SD, mmHg		10 219	78.7 ± 10.2	539	79.3 ± 10.2
Hypertension, No. (%)		10 119		272	
Yes			2523 (24.9)		73 (26.8)
No			7596 (75.1)		199 (73.2)
Educational level, No. (%)		10 222		255	
Unfinished primary school			39 (0.4)		2 (0.8)
Primary school			940 (9.2)		32 (12.6)
Secondary school			5088 (49.8)		131 (51.4)
University degree			4155 (40.7)		90 (35.3)
Coronary stenosis, No. (%)		9110		436	
0%			4167 (45.7)		171 (39.2)
1–49%			4159 (45.7)		204 (46.8)
≥50%			784 (8.6)		61 (14.0)
CAC, No. (%)		8925		489	
0 Agatston units			4282 (48.0)		194 (39.7)
1–99 Agatston units			3080 (34.5)		189 (38.7)
≥100 Agatston units			1563 (17.5)		106 (21.7)
Carotid plaque, No. (%)		10 205		513	
No plaque			4025 (39.4)		193 (37.6)
Unilateral plaque/s			3114 (30.5)		161 (31.4)
Bilateral plaques			3066 (30.0)		159 (31.0)

Total population refers to male adolescents in conscription database with data in SCAPIS.

BMI: body mass index, BP: blood pressure, CAC: coronary artery calcium, DBP: diastolic blood pressure, MAP: mean arterial pressure, PP: pulse pressure, SBP: systolic blood pressure, SCAPIS: Swedish CArdioPulmonary bioImage Study.

© 2025 Herraiz-Adillo Á et al. *JAMA Cardiology*.

eTable 2. Multinomial Logistic Regression, Adjusted Prevalences for the Association of 2025 ACC/AHA Blood Pressure Categories in Adolescence With Coronary and Carotid Atherosclerosis in Middle Age[2]

2025 ACC/AHA BP classification	Adjusted prevalence (95% CI)		Adjusted prevalence (95% CI)		Adjusted prevalence (95% CI)	
	No coronary stenosis		1-49% coronary stenosis		≥50% coronary stenosis	
**Normal BP**	48.1	(45.8-50.5)	45.0	(42.6-47.3)	6.9	(5.7-8.1)
**Elevated BP**	46.9	(45.1-48.7)	44.5	(42.7-46.3)	8.6	(7.6-9.7)
**Hypertension stage 1**	46.1	(44.4-47.8)	45.1	(43.4-46.8)	8.7	(7.8-9.7)
**Hypertension stage 2**	40.3	(37.9-42.7)	49.7	(47.2-52.2)	10.1	(8.6-11.5)
	CAC score = 0		CAC score 1-99		CAC score ≥100	
**Normal BP**	50.6	(48.3-53.0)	34.7	(32.4-37.0)	14.7	(13.0-16.3)
**Elevated BP**	48.9	(47.1-50.7)	33.8	(32.0-35.6)	17.3	(15.9-18.7)
**Hypertension stage 1**	48.5	(46.8-50.2)	33.5	(31.8-35.1)	18.0	(16.7-19.3)
**Hypertension stage 2**	42.2	(39.8-44.6)	37.9	(35.4-40.3)	19.9	(18.0-21.9)
	No carotid plaque		Unilateral carotid plaque/s		Bilateral carotid plaques	
**Normal BP**	42.4	(40.2-44.6)	31.0	(28.9-33.1)	26.6	(24.7-28.6)
**Elevated BP**	41.2	(39.5-42.9)	29.2	(27.6-30.9)	29.6	(28.0-31.2)
**Hypertension stage 1**	37.9	(36.3-39.5)	31.5	(29.9-33.0)	30.6	(29.1-32.1)
**Hypertension stage 2**	36.2	(34.0-38.5)	30.3	(28.1-32.5)	33.5	(31.3-35.7)

Marginal prevalences are adjusted for age at conscription, age at SCAPIS, site in conscription, site in SCAPIS, conscription year, BMI at conscription (linear and quadratic terms), smoking at conscription, and education level at SCAPIS.
Normal BP: SBP <120 and DBP <80 mmHg; elevated BP: SBP =120-129 and DBP <80 mmHg; hypertension stage 1: SBP =130-139 or DBP =80-89 mmHg; hypertension stage 2: SBP ≥140 or DBP ≥90 mmHg.
ACC/AHA: American College of Cardiology/American Heart Association, BMI: body mass index, BP: blood pressure, CAC: coronary artery calcium, CI: confidence interval, SCAPIS: Swedish CArdioPulmonary bioImage Study.

© 2025 Herraiz-Adillo Á et al. *JAMA Cardiology*.

eTable 3. Multinomial Logistic Models, Adjusted Prevalences for the Association of 2025 ACC/AHA Blood Pressure Categories in Adolescence With Coronary Stenosis in Middle Age in the 11 Most Relevant Coronary Segments[2]

		Relevant coronary segments										
	**2025 ACC/AHA BP classification**	**1**	**2**	**3**	**5**	**6**	**7**	**9**	**11**	**12**	**13**	**17**
**Coronary stenosis (1-49%)**	**Normal**	14.4 (12.7, 16.0)	12.3 (10.7, 13.8)	8.1 (6.8, 9.5)	12.9 (11.3, 14.5)	35.6 (33.4, 37.9)	23.8 (21.8, 25.8)	7.4 (6.1, 8.7)	12.2 (10.7, 13.8)	5.3 (4.2, 6.4)	4.0 (3.0, 5.0)	3.6 (2.7, 4.5)
	**Elevated**	15.2 (13.9, 16.6)	14.4 (13.1, 15.8)	9.6 (8.4, 10.7)	13.6 (12.3, 14.8)	36.1 (34.4, 37.9)	25.4 (23.8, 27.1)	9.0 (7.8, 10.1)	14.5 (13.2, 15.8)	6.4 (5.5, 7.4)	5.3 (4.4, 6.1)	4.2 (3.4, 5.0)
	**Hypertension stage 1**	15.8 (14.6, 17.0)	15.6 (14.3, 16.8)	10.8 (9.7, 11.8)	12.9 (11.7, 14.0)	36.7 (35.1, 38.4)	27.6 (26.1, 29.2)	9.5 (8.4, 10.5)	14.7 (13.5, 15.9)	6.3 (5.4, 7.1)	5.7 (4.9, 6.5)	3.8 (3.1, 4.4)
	**Hypertension stage 2**	19.4 (17.5, 21.4)	16.8 (15.0, 18.7)	12.2 (10.6, 13.8)	14.4 (12.7, 16.1)	40.7 (38.3, 43.1)	31.3 (29.0, 33.6)	11.5 (9.8, 13.1)	19.3 (17.4, 21.2)	8.3 (6.9, 9.7)	7.0 (5.7, 8.2)	4.4 (3.4, 5.4)
**Coronary stenosis (≥50%)**	**Normal**	0.6 (0.2, 1.0)	0.7 (0.3, 1.1)	0.7 (0.3, 1.1)	0.1 (-0.1, 0.2)	1.8 (1.1, 2.4)	3.6 (2.6, 4.5)	1.5 (0.9, 2.1)	0.5 (0.2, 0.9)	0.6 (0.2, 0.9)	0.3 (0.0, 0.5)	0.5 (0.1, 0.8)
	**Elevated**	0.7 (0.4, 1.1)	1.1 (0.7, 1.5)	0.6 (0.3, 0.9)	0.2 (0.0, 0.4)	3.1 (2.4, 3.7)	4.1 (3.4, 4.9)	1.3 (0.9, 1.8)	0.8 (0.4, 1.1)	0.4 (0.1, 0.6)	0.5 (0.2, 0.8)	0.6 (0.3, 0.9)
	**Hypertension stage 1**	1.1 (0.7, 1.4)	1.3 (0.9, 1.7)	0.5 (0.3, 0.7)	0.2 (0.0, 0.3)	3.1 (2.5, 3.7)	3.9 (3.2, 4.5)	1.7 (1.2, 2.1)	0.9 (0.6, 1.2)	0.6 (0.3, 0.8)	0.6 (0.3, 0.9)	0.5 (0.3, 0.7)
	**Hypertension stage 2**	1.4 (0.8, 2.0)	1.7 (1.1, 2.4)	0.9 (0.4, 1.4)	0.3 (0.0, 0.6)	3.7 (2.7, 4.6)	4.9 (3.8, 5.9)	1.8 (1.1, 2.5)	1.1 (0.6, 1.6)	1.0 (0.5, 1.5)	1.0 (0.5, 1.5)	0.5 (0.1, 0.9)

Values refer to marginal prevalences with 95% confidence intervals adjusted for age at conscription, age at SCAPIS, site in conscription, site in SCAPIS, conscription year, BMI at conscription (linear and quadratic terms), smoking at conscription, and education level at SCAPIS.
Coronary segments are named in accordance with the Society of Cardiovascular Computed Tomography guidelines.[6]
Normal BP: SBP <120 and DBP <80 mmHg; elevated BP: SBP =120-129 and DBP <80 mmHg; hypertension stage 1: SBP =130-139 or DBP =80-89 mmHg; hypertension stage 2: SBP ≥140 or DBP ≥90 mmHg.
ACC/AHA: American College of Cardiology/American Heart Association, BMI: body mass index, BP: blood pressure, SCAPIS: Swedish CArdioPulmonary bioImage Study.

© 2025 Herraiz-Adillo Á et al. *JAMA Cardiology*.

eTable 4. Multinomial Logistic Regression, Odds Ratios for the Association of 2025 ACC/AHA Systolic Blood Pressure Categories in Adolescence With Coronary and Carotid Atherosclerosis in Middle Age[2]

	Basic adjustment			Extended adjustment		
**2025 ACC/AHA SBP classification**	**OR**	**(95% CI)**	**P**	**OR**	**(95% CI)**	**P**
**Coronary stenosis**						
**1-49%**						
**<120, ref.**	1.00	-	-	1.00	-	-
**120-129**	1.08	(0.95-1.22)	0.23	1.05	(0.93-1.19)	0.42
**130-139**	1.15	(1.01-1.31)	0.03	1.11	(0.97-1.26)	0.13
**≥140**	1.47	(1.27-1.71)	<0.001	1.40	(1.20-1.63)	<0.001
**≥50%**						
**<120, ref.**	1.00	-	-	1.00	-	-
**120-129**	1.51	(1.19-1.91)	0.001	1.47	(1.15-1.86)	0.002
**130-139**	1.49	(1.17-1.90)	0.001	1.43	(1.12-1.83)	0.005
**≥140**	2.08	(1.59-2.73)	<0.001	1.97	(1.50-2.60)	<0.001
**CAC score**						
**1-99**						
**<120, ref.**	1.00	-	-	1.00	-	-
**120-129**	1.06	(0.93-1.21)	0.37	1.05	(0.92-1.19)	0.51
**130-139**	1.11	(0.97-1.28)	0.12	1.08	(0.94-1.24)	0.26
**≥140**	1.47	(1.25-1.72)	<0.001	1.41	(1.20-1.66)	<0.001
**≥100**						
**<120, ref.**	1.00	-	-	1.00	-	-
**120-129**	1.40	(1.17-1.67)	<0.001	1.33	(1.11-1.60)	0.002
**130-139**	1.54	(1.29-1.85)	<0.001	1.44	(1.19-1.73)	<0.001
**≥140**	1.96	(1.59-2.41)	<0.001	1.78	(1.44-2.20)	<0.001
**Ultrasound carotid plaque**						
**Unilateral**						
**<120, ref.**	1.00	-	-	1.00	-	-
**120-129**	0.95	(0.83-1.08)	0.45	0.95	(0.84-1.09)	0.49
**130-139**	1.15	(1.01-1.32)	0.04	1.17	(1.02-1.34)	0.03
**≥140**	1.14	(0.97-1.34)	0.11	1.16	(0.98-1.36)	0.08
**Bilateral**						
**<120, ref.**	1.00	-	-	1.00	-	-
**120-129**	1.08	(0.94-1.24)	0.25	1.10	(0.96-1.27)	0.16
**130-139**	1.34	(1.16-1.55)	<0.001	1.39	(1.20-1.60)	<0.001
**≥140**	1.45	(1.23-1.71)	<0.001	1.53	(1.29-1.81)	<0.001

Reference category for CCTA stenosis, CAC score and carotid plaques: no stenosis, CAC score = 0 Agatston units and no plaque, respectively.
Basic adjusted model: adjusted for age at conscription, age at SCAPIS, site in conscription, site in SCAPIS, and conscription year.
Extended adjusted model: basic adjustment plus BMI at conscription (linear and quadratic terms), smoking at conscription, and education level at SCAPIS.
ACC/AHA: American College of Cardiology/American Heart Association, BMI: body mass index, CAC: coronary artery calcium, CI: confidence interval, OR: odds ratio, ref.: reference, SBP: systolic blood pressure, SCAPIS: Swedish CArdioPulmonary bioImage Study.

© 2025 Herraiz-Adillo Á et al. *JAMA Cardiology*.

eTable 5. Multinomial Logistic Regression, Adjusted Prevalences for the Association of 2025 ACC/AHA Systolic Blood Pressure Categories in Adolescence With Coronary and Carotid Atherosclerosis in Middle Age[2]

2025 ACC/AHA SBP classification	Adjusted prevalence (95% CI)		Adjusted prevalence (95% CI)		Adjusted prevalence (95% CI)	
	**No coronary stenosis**		**1-49% coronary stenosis**		**≥50% coronary stenosis**	
**<120**	48.8	(46.6-51.0)	44.6	(42.3-46.9)	6.6	(5.4-7.7)
**120-129**	46.6	(44.9-48.3)	44.4	(42.7-46.1)	9.0	(8.0-10.0)
**130-139**	45.7	(43.9-47.5)	45.7	(43.8-47.5)	8.6	(7.6-9.6)
**≥140**	40.1	(37.7-42.6)	49.7	(47.2-52.3)	10.1	(8.6-11.6)
	**CAC score = 0**		**CAC score 1-99**		**CAC score ≥100**	
**<120**	51.5	(49.2-53.8)	34.2	(32.0-36.4)	14.3	(12.7-15.9)
**120-129**	48.9	(47.2-50.6)	33.5	(31.9-35.2)	17.6	(16.3-18.9)
**130-139**	47.8	(45.9-49.6)	33.8	(32.0-35.6)	18.4	(17.0-19.8)
**≥140**	42.1	(39.6-44.6)	38.3	(35.8-40.9)	19.6	(17.6-21.5)
	**No carotid plaque**		**Unilateral carotid plaque/s**		**Bilateral carotid plaques**	
**<120**	42.2	(40.1-44.3)	31.2	(29.1-33.2)	26.6	(24.7-28.5)
**120-129**	41.7	(40.1-43.3)	29.3	(27.8-30.8)	28.9	(27.5-30.4)
**130-139**	36.9	(35.2-38.6)	31.5	(29.9-33.2)	31.6	(30.0-33.2)
**≥140**	35.9	(33.6-38.2)	30.4	(28.1-32.6)	33.8	(31.5-36.0)

Marginal prevalences are adjusted for age at conscription, age at SCAPIS, site in conscription, site in SCAPIS, conscription year, BMI at conscription (linear and quadratic terms), smoking at conscription, and education level at SCAPIS.
Normal BP: SBP <120 and DBP <80 mmHg; elevated BP: SBP =120-129 and DBP <80 mmHg; hypertension stage 1: SBP =130-139 or DBP =80-89 mmHg; hypertension stage 2: SBP ≥140 or DBP ≥90 mmHg.
ACC/AHA: American College of Cardiology/American Heart Association, BMI: body mass index, CAC: coronary artery calcium, CI: confidence interval, SBP: systolic blood pressure, SCAPIS: Swedish CArdioPulmonary bioImage Study.

© 2025 Herraiz-Adillo Á et al. *JAMA Cardiology*.

eTable 6. Multinomial Logistic Models, Adjusted Prevalences for the Association of 2025 ACC/AHA Systolic Blood Pressure Categories in Adolescence With Coronary Stenosis in Middle Age in the 11 Most Relevant Coronary Segments[2]

		Relevant coronary segments										
	**2025 ACC/AHA SBP classification**	**1**	**2**	**3**	**5**	**6**	**7**	**9**	**11**	**12**	**13**	**17**
**Coronary stenosis (1-49%)**	**<120**	14.2 (12.6, 15.8)	12.1 (10.6, 13.6)	8.1 (6.8, 9.4)	12.7 (11.2, 14.2)	35.3 (33.1, 37.4)	23.8 (21.8, 25.7)	7.3 (6.1, 8.6)	12.1 (10.6, 13.6)	5.0 (4.0, 6.1)	4.1 (3.1, 5.0)	3.4 (2.5, 4.2)
	**120-129**	15.6 (14.3, 16.8)	14.6 (13.4, 15.8)	9.9 (8.8, 10.9)	13.5 (12.3, 14.7)	36.2 (34.6, 37.9)	25.8 (24.3, 27.3)	9.0 (8.0, 10.1)	14.4 (13.1, 15.6)	6.6 (5.7, 7.5)	5.3 (4.5, 6.2)	4.1 (3.4, 4.9)
	**130-139**	15.9 (14.6, 17.2)	16.0 (14.6, 17.3)	10.9 (9.7, 12.0)	13.2 (12.0, 14.5)	37.0 (35.2, 38.8)	27.8 (26.1, 29.4)	9.5 (8.4, 10.7)	15.4 (14.0, 16.7)	6.5 (5.5, 7.4)	5.8 (4.9, 6.7)	3.9 (3.2, 4.7)
	**≥140**	19.3 (17.4, 21.3)	16.6 (14.8, 18.5)	12.1 (10.4, 13.8)	14.1 (12.3, 15.8)	40.8 (38.3, 43.3)	31.3 (28.9, 33.6)	11.6 (10.0, 13.3)	19.1 (17.1, 21.1)	8.1 (6.7, 9.6)	7.0 (5.7, 8.3)	4.5 (3.4, 5.5)
**Coronary stenosis (≥50%)**	**<120**	0.6 (0.3, 1.0)	0.7 (0.3, 1.1)	0.6 (0.3, 1.0)	0.1 (0.0, 0.2)	1.6 (1.0, 2.2)	3.4 (2.6, 4.3)	1.3 (0.8, 1.9)	0.5 (0.2, 0.8)	0.5 (0.2, 0.9)	0.2 (0.0, 0.5)	0.4 (0.1, 0.7)
	**120-129**	0.9 (0.5, 1.2)	1.3 (0.9, 1.7)	0.6 (0.3, 0.9)	0.2 (0.1, 0.4)	3.3 (2.6, 3.9)	4.1 (3.4, 4.8)	1.7 (1.3, 2.2)	1.0 (0.6, 1.3)	0.4 (0.2, 0.7)	0.5 (0.3, 0.8)	0.7 (0.4, 1.0)
	**130-139**	1.1 (0.7, 1.5)	1.1 (0.7, 1.5)	0.6 (0.3, 0.9)	0.2 (0.0, 0.3)	3.1 (2.5, 3.8)	3.9 (3.2, 4.7)	1.4 (0.9, 1.8)	0.7 (0.4, 1.0)	0.6 (0.3, 0.9)	0.7 (0.4, 1.0)	0.4 (0.2, 0.7)
	**≥140**	1.3 (0.7, 1.8)	1.8 (1.1, 2.5)	0.9 (0.4, 1.3)	0.3 (0.0, 0.6)	3.5 (2.6, 4.5)	4.9 (3.8, 6.0)	1.8 (1.1, 2.5)	1.1 (0.6, 1.7)	1.0 (0.5, 1.5)	1.0 (0.5, 1.5)	0.5 (0.1, 0.9)

Values refer to marginal prevalences with 95% confidence intervals adjusted for age at conscription, age at SCAPIS, site in conscription, site in SCAPIS, conscription year, BMI at conscription (linear and quadratic terms), smoking at conscription, and education level at SCAPIS.
Coronary segments are named in accordance with the Society of Cardiovascular Computed Tomography guidelines.[6]
Normal BP: SBP <120 and DBP <80 mmHg; elevated BP: SBP =120-129 and DBP <80 mmHg; hypertension stage 1: SBP =130-139 or DBP =80-89 mmHg; hypertension stage 2: SBP ≥140 or DBP ≥90 mmHg.
ACC/AHA: American College of Cardiology/American Heart Association, BMI: body mass index, SBP: systolic blood pressure, SCAPIS: Swedish CArdioPulmonary bioImage Study.

© 2025 Herraiz-Adillo Á et al. *JAMA Cardiology*.

eTable 7. Multinomial Logistic Regression, Odds Ratios for the Association of 2025 ACC/AHA Diastolic Blood Pressure Categories in Adolescence With Coronary and Carotid Atherosclerosis in Middle Age[2]

	Basic adjustment			Extended adjustment		
**2025 ACC/AHA DBP classification**	**OR**	**(95% CI)**	**P**	**OR**	**(95% CI)**	**P**
**Coronary stenosis**						
**1-49%**						
**<80, ref.**	1.00	-	-	1.00	-	-
**80-89**	1.08	(0.95-1.23)	0.26	1.05	(0.92-1.20)	0.47
**≥90**	1.37	(0.89-2.11)	0.15	1.35	(0.88-2.08)	0.17
**≥50%**						
**<80, ref.**	1.00	-	-	1.00	-	-
**80-89**	1.37	(1.11-1.68)	0.003	1.32	(1.07-1.63)	0.009
**≥90**	1.05	(0.51-2.16)	0.90	1.06	(0.51-2.19)	0.87
**CAC score**						
**1-99**						
**<80, ref.**	1.00	-	-	1.00	-	-
**80-89**	1.02	(0.88-1.17)	0.82	0.99	(0.86-1.14)	0.91
**≥90**	1.09	(0.67-1.76)	0.73	1.07	(0.66-1.74)	0.78
**≥100**						
**<80, ref.**	1.00	-	-	1.00	-	-
**80-89**	1.25	(1.06-1.48)	0.009	1.18	(1.00-1.40)	0.06
**≥90**	1.82	(1.11-2.99)	0.02	1.80	(1.09-2.97)	0.02
**Ultrasound carotid plaque**						
**Unilateral**						
**<80, ref.**	1.00	-	-	1.00	-	-
**80-89**	0.97	(0.84-1.11)	0.62	0.97	(0.84-1.12)	0.69
**≥90**	1.01	(0.64-1.59)	0.98	1.05	(0.66-1.66)	0.85
**Bilateral**						
**<80, ref.**	1.00	-	-	1.00	-	-
**80-89**	1.00	(0.87-1.15)	0.98	1.01	(0.88-1.17)	0.86
**≥90**	1.21	(0.79-1.86)	0.39	1.28	(0.83-1.97)	0.27

Reference category for CCTA stenosis, CAC score and carotid plaques: no stenosis, CAC score = 0 Agatston units and no plaque, respectively.
Basic adjusted model: adjusted for age at conscription, age at SCAPIS, site in conscription, site in SCAPIS, and conscription year.
Extended adjusted model: basic adjustment plus BMI at conscription (linear and quadratic terms), smoking at conscription, and education level at SCAPIS.
Normal BP: SBP <120 and DBP <80 mmHg; elevated BP: SBP =120-129 and DBP <80 mmHg; hypertension stage 1: SBP =130-139 or DBP =80-89 mmHg; hypertension stage 2: SBP ≥140 or DBP ≥90 mmHg.
ACC/AHA: American College of Cardiology/American Heart Association, BMI: body mass index, CAC: coronary artery calcium, CI: confidence interval, DBP: diastolic blood pressure, OR: odds ratio, ref.: reference, SCAPIS: Swedish CArdioPulmonary bioImage Study.

© 2025 Herraiz-Adillo Á et al. *JAMA Cardiology*.

eTable 8. Multinomial Logistic Regression, Adjusted Prevalences for the Association of 2025 ACC/AHA Diastolic Blood Pressure Categories in Adolescence With Coronary and Carotid Atherosclerosis in Middle Age[2]

2025 ACC/AHA DBP classification	Adjusted prevalence (95% CI)		Adjusted prevalence (95% CI)		Adjusted prevalence (95% CI)	
	No coronary stenosis		1-49% coronary stenosis		≥50% coronary stenosis	
**<80**	46.1	(45.0-47.2)	45.6	(44.5-46.7)	8.3	(7.7-8.9)
**80-89**	44.2	(41.4-46.9)	45.5	(42.8-48.3)	10.3	(8.8-11.9)
**≥90**	40.0	(30.6-49.3)	52.6	(43.1-62.1)	7.4	(3.0-11.9)
	CAC score = 0		CAC score 1-99		CAC score ≥100	
**<80**	48.2	(47.1-49.3)	34.8	(33.7-35.8)	17.0	(16.2-17.9)
**80-89**	47.1	(44.4-49.9)	33.5	(30.9-36.2)	19.4	(17.3-21.4)
**≥90**	42.5	(33.0-52.0)	32.0	(23.0-41.0)	25.5	(18.0-32.9)
	No carotid plaque		Unilateral carotid plaque/s		Bilateral carotid plaques	
**<80**	39.5	(38.4-40.5)	30.6	(29.7-31.6)	29.9	(29.0-30.9)
**80-89**	39.7	(37.1-42.2)	29.9	(27.5-32.3)	30.4	(28.1-32.8)
**≥90**	36.2	(27.7-44.8)	29.2	(21.2-37.3)	34.5	(26.7-42.4)

Marginal prevalences are adjusted for age at conscription, age at SCAPIS, site in conscription, site in SCAPIS, conscription year, BMI at conscription (linear and quadratic terms), smoking at conscription, and education level at SCAPIS.
Normal BP: SBP <120 and DBP <80 mmHg; elevated BP: SBP =120-129 and DBP <80 mmHg; hypertension stage 1: SBP =130-139 or DBP =80-89 mmHg; hypertension stage 2: SBP ≥140 or DBP ≥90 mmHg.
ACC/AHA: American College of Cardiology/American Heart Association, BMI: body mass index, CAC: coronary artery calcium, CI: confidence interval, DBP: diastolic blood pressure, SCAPIS: Swedish CArdioPulmonary bioImage Study.

© 2025 Herraiz-Adillo Á et al. *JAMA Cardiology*.

eTable 9. Multinomial Logistic Models, Adjusted Prevalences for the Association of 2025 ACC/AHA Diastolic Blood Pressure Categories in Adolescence With Coronary Stenosis in Middle Age in the 11 Most Relevant Coronary Segments[2]

		Relevant coronary segments										
	**2025 ACC/AHA DBP classification**	**1**	**2**	**3**	**5**	**6**	**7**	**9**	**11**	**12**	**13**	**17**
**Coronary stenosis (1-49%)**	**<80**	15.7 (14.9, 16.5)	14.7 (13.9, 15.5)	9.8 (9.1, 10.5)	13.2 (-, -)	37.0 (35.9, 38.0)	26.3 (25.3, 27.2)	9.3 (8.6, 9.9)	14.6 (13.9, 15.4)	6.4 (5.9, 7.0)	5.2 (4.7, 5.7)	4.2 (3.7, 4.6)
	**80-89**	17.4 (15.5, 19.4)	15.6 (13.7, 17.5)	11.8 (10.1, 13.5)	13.3 (-, -)	36.7 (34.1, 39.3)	30.2 (27.7, 32.6)	9.6 (8.0, 11.2)	16.1 (14.2, 18.0)	6.5 (5.2, 7.8)	6.9 (5.6, 8.3)	3.0 (2.1, 3.9)
	**≥90**	19.3 (12.6, 26.1)	17.8 (11.1, 24.6)	14.1 (8.0, 20.2)	20.9 (-, -)	43.9 (34.7, 53.1)	30.1 (21.8, 38.3)	8.0 (3.2, 12.8)	22.2 (15.0, 29.4)	10.9 (5.5, 16.3)	8.3 (3.6, 13.1)	4.4 (0.9, 7.9)
**Coronary stenosis (≥50%)**	**<80**	0.9 (0.7, 1.1)	1.1 (0.8, 1.3)	0.6 (0.4, 0.8)	0.2 (-, -)	2.7 (2.3, 3.1)	4.0 (3.5, 4.4)	1.3 (1.1, 1.6)	0.8 (0.6, 1.0)	0.5 (0.3, 0.7)	0.6 (0.4, 0.8)	0.5 (0.3, 0.7)
	**80-89**	1.2 (0.6, 1.7)	1.9 (1.2, 2.7)	0.7 (0.3, 1.1)	0.3 (-, -)	4.0 (3.0, 5.0)	4.5 (3.4, 5.5)	2.7 (1.9, 3.6)	1.1 (0.6, 1.6)	0.9 (0.5, 1.4)	0.5 (0.2, 0.9)	0.8 (0.3, 1.2)
	**≥90**	2.1 (-0.3, 4.6)	0.9 (-0.9, 2.7)	0.8 (-0.8, 2.3)	0.0 (-, -)	4.6 (1.0, 8.2)	3.6 (0.5, 6.7)	0.8 (-0.8, 2.4)	0.0 (0.0, 0.0)	1.4 (-0.5, 3.2)	0.8 (-0.7, 2.3)	0.0 (0.0, 0.0)

Values refer to marginal prevalences with 95% confidence intervals adjusted for age at conscription, age at SCAPIS, site in conscription, site in SCAPIS, conscription year, BMI at conscription (linear and quadratic terms), smoking at conscription, and education level at SCAPIS.
Coronary segments are named in accordance with the Society of Cardiovascular Computed Tomography guidelines.[6]
Normal BP: SBP <120 and DBP <80 mmHg; elevated BP: SBP =120-129 and DBP <80 mmHg; hypertension stage 1: SBP =130-139 or DBP =80-89 mmHg; hypertension stage 2: SBP ≥140 or DBP ≥90 mmHg.
ACC/AHA: American College of Cardiology/American Heart Association, BMI: body mass index, DBP: diastolic blood pressure, SCAPIS: Swedish CArdioPulmonary bioImage Study.

© 2025 Herraiz-Adillo Á et al. *JAMA Cardiology*.

eTable 10. Associations of 2025 ACC/AHA Blood Pressure Categories in Adolescence With Coronary Atherosclerosis in Middle Age Considering Additional Binomial Atherosclerosis Categories

	aOR	(95% CI)	P	Adjusted prevalence	(95% CI)
**Any coronary stenosis (≥1%)**					
**Normal BP, ref.**	1.00	-	-	51.9	(49.5-54.2)
**Elevated BP**	1.06	(0.93-1.20)	0.39	53.1	(51.3-54.9)
**Hypertension stage 1**	1.09	(0.96-1.24)	0.16	53.9	(52.2-55.6)
**Hypertension stage 2**	1.42	(1.22-1.64)	<0.001	59.7	(57.3-62.1)
**Any significant coronary stenosis (≥50%)**					
**Normal BP, ref.**	1.00	-	-	6.9	(5.7-8.1)
**Elevated BP**	1.29	(1.02-1.64)	0.04	8.6	(7.6-9.7)
**Hypertension stage 1**	1.31	(1.04-1.66)	0.02	8.8	(7.8-9.7)
**Hypertension stage 2**	1.54	(1.19-2.00)	0.001	10.1	(8.6-11.5)
**Any non-calcified plaque**					
**Normal BP, ref.**	1.00	-	-	10.5	(9.0-11.9)
**Elevated BP**	1.01	(0.82-1.23)	0.95	10.5	(9.4-11.7)
**Hypertension stage 1**	1.10	(0.90-1.33)	0.36	11.3	(10.3-12.4)
**Hypertension stage 2**	1.32	(1.06-1.65)	0.01	13.3	(11.6-14.9)
**SIS score ≥4**					
**Normal BP, ref.**	1.00	-	-	14.7	(13.0-16.3)
**Elevated BP**	1.28	(1.08-1.53)	0.005	17.8	(16.4-19.3)
**Hypertension stage 1**	1.43	(1.21-1.69)	<0.001	19.4	(18.0-20.7)
**Hypertension stage 2**	1.80	(1.48-2.17)	<0.001	22.9	(20.8-24.9)
**CAC score ≥100 Agatston units**					
**Normal BP, ref.**	1.00	-	-	14.7	(13.0-16.3)
**Elevated BP**	1.23	(1.03-1.47)	0.02	17.3	(15.9-18.7)
**Hypertension stage 1**	1.30	(1.10-1.55)	0.003	18.0	(16.7-19.3)
**Hypertension stage 2**	1.49	(1.23-1.82)	<0.001	19.9	(18.0-21.9)

Binomial logistic models depicting aORs and prevalences are adjusted for age at conscription, age at SCAPIS, site in conscription, site in SCAPIS, conscription year, BMI at conscription (linear and quadratic terms), smoking at conscription, and education level at SCAPIS.
Normal BP: SBP <120 and DBP <80 mmHg; elevated BP: SBP =120-129 and DBP <80 mmHg; hypertension stage 1: SBP =130-139 or DBP =80-89 mmHg; hypertension stage 2: SBP ≥140 or DBP ≥90 mmHg.
ACC/AHA: American College of Cardiology/American Heart Association, aOR: odds ratio, BMI: body mass index, BP: blood pressure, CAC: coronary artery calcium, CI: confidence interval, CT: Computed Tomography, ref.: reference, SCAPIS: Swedish CArdioPulmonary bioImage Study, SIS: segment involvement score.[8]

© 2025 Herraiz-Adillo Á et al. *JAMA Cardiology*.

eTable 11. Sensitivity Analysis, Associations of 2025 ACC/AHA Blood Pressure Categories in Adolescence With Coronary Stenosis in Middle Age Considering Influential Factors for Defining Coronary Stenosis[2]

	Considering participants with data in any of the 11 relevant coronary segments (Ref. analysis) n = 9110			Considering participants with data in all of the 11 relevant coronary segments n = 7572			Considering participants with data in any of the 18 coronary segments n = 9110			Considering calcium blooming as ≥50% stenosis n = 9110			Exclusion of participants with stents in the 11 relevant coronary segments n = 9013		
	**OR**	**(95% CI)**	**P**	**OR**	**(95% CI)**	**P**	**OR**	**(95% CI)**	**P**	**OR**	**(95% CI)**	**P**	**OR**	**(95% CI)**	**P**
**2025 ACC/AHA BP classification**															
**Coronary stenosis**															
**1-49%**															
**Normal BP, ref.**	1.00	-	-	1.00	-	-	1.00	-	-	1.00	-	-	1.00	-	-
**Elevated BP**	1.02	(0.90-1.17)	0.73	1.07	(0.92-1.23)	0.38	1.02	(0.89-1.16)	0.80	1.00	(0.88-1.15)	0.98	1.02	(0.90-1.17)	0.73
**Hypertension stage 1**	1.06	(0.93-1.20)	0.40	1.05	(0.91-1.21)	0.49	1.06	(0.93-1.20)	0.40	1.02	(0.89-1.16)	0.77	1.06	(0.93-1.20)	0.40
**Hypertension stage 2**	1.36	(1.16-1.58)	<0.001	1.39	(1.17-1.65)	<0.001	1.36	(1.16-1.58)	<0.001	1.30	(1.11-1.52)	0.001	1.36	(1.16-1.58)	<0.001
**≥ 50%**															
**Normal BP, ref.**	1.00	-	-	1.00	-	-	1.00	-	-	1.00	-	-	1.00	-	-
**Elevated BP**	1.31	(1.02-1.68)	0.03	1.14	(0.84-1.55)	0.39	1.34	(1.05-1.72)	0.02	1.30	(1.05-1.60)	0.01	1.28	(0.98-1.66)	0.07
**Hypertension stage 1**	1.35	(1.06-1.72)	0.01	1.40	(1.05-1.86)	0.02	1.36	(1.07-1.73)	0.01	1.40	(1.15-1.72)	0.001	1.25	(0.97-1.61)	0.09
**Hypertension stage 2**	1.84	(1.40-2.42)	<0.001	1.58	(1.13-2.22)	0.007	1.89	(1.44-2.48)	<0.001	1.93	(1.53-2.43)	<0.001	1.81	(1.36-2.41)	<0.001

Reference category for CCTA stenosis: no stenosis.
All models refer to the extended adjusted model: adjusted for age at conscription, age at SCAPIS, site in conscription, site in SCAPIS, conscription year, BMI at conscription (linear and quadratic terms), smoking at conscription, and education level at SCAPIS.
Normal BP: SBP <120 and DBP <80 mmHg; elevated BP: SBP =120-129 and DBP <80 mmHg; hypertension stage 1: SBP =130-139 or DBP =80-89 mmHg; hypertension stage 2: SBP ≥140 or DBP ≥90 mmHg.
ACC/AHA: American College of Cardiology/American Heart Association, BMI: body mass index, BP: blood pressure, CI: confidence interval, CVD: cardiovascular disease, OR: odds ratio, ref.: reference, SCAPIS: Swedish CArdioPulmonary bioImage Study.

© 2025 Herraiz-Adillo Á et al. *JAMA Cardiology*.

eTable 12. Sensitivity Analysis, Associations of 2025 ACC/AHA Blood Pressure Categories in Adolescence With Coronary Stenosis in Middle Age Considering Additional Levels of Adjustment and Excluding Participants With Cardiovascular Disease[2]

	Considering the 11 relevant coronary segments (Ref. analysis) n = 9110			Extended model without BMI at conscription n = 9110			Extended model plus cardiorespiratory fitness at conscription n = 9110			Extended model plus cardiorespiratory fitness and muscular strength at conscription n = 9013			Extended model plus family history of myocardial infarction and stroke n = 8769			Exclusion of CVD n = 8775		
	**OR**	**(95% CI)**	**P**	**OR**	**(95% CI)**	**P**	**OR**	**(95% CI)**	**P**	**OR**	**(95% CI)**	**P**	**OR**	**(95% CI)**	**P**	**OR**	**(95% CI)**	**P**
**2025 ACC/AHA BP classification**																		
**Coronary stenosis**																		
**1-49%**																		
**Normal BP, ref.**	1.00	-	-	1.00	-	-	1.00	-	-	1.00	-	-	1.00	-	-	1.00	-	-
**Elevated BP**	1.02	(0.90-1.17)	0.73	1.06	(0.93-1.21)	0.37	1.04	(0.91-1.20)	0.55	1.05	(0.91-1.20)	0.53	1.03	(0.90-1.18)	0.64	1.03	(0.90-1.18)	0.64
**Hypertension stage 1**	1.06	(0.93-1.20)	0.40	1.12	(0.99-1.27)	0.08	1.07	(0.93-1.23)	0.35	1.07	(0.93-1.23)	0.32	1.08	(0.94-1.23)	0.27	1.06	(0.93-1.21)	0.38
**Hypertension stage 2**	1.36	(1.16-1.58)	<0.001	1.48	(1.27-1.72)	<0.001	1.43	(1.21-1.68)	<0.001	1.43	(1.22-1.69)	<0.001	1.36	(1.16-1.59)	<0.001	1.35	(1.15-1.57)	<0.001
**≥ 50%**																		
**Normal BP, ref.**	1.00	-	-	1.00	-	-	1.00	-	-	1.00	-	-	1.00	-	-	1.00	-	-
**Elevated BP**	1.31	(1.02-1.68)	0.03	1.37	(1.07-1.76)	0.01	1.35	(1.02-1.78)	0.04	1.36	(1.03-1.80)	0.03	1.36	(1.05-1.76)	0.02	1.24	(0.95-1.62)	0.11
**Hypertension stage 1**	1.35	(1.06-1.72)	0.01	1.46	(1.15-1.86)	0.002	1.39	(1.06-1.83)	0.02	1.42	(1.08-1.86)	0.01	1.41	(1.10-1.81)	0.007	1.20	(0.93-1.56)	0.17
**Hypertension stage 2**	1.84	(1.40-2.42)	<0.001	2.07	(1.58-2.72)	<0.001	1.88	(1.38-2.56)	<0.001	1.91	(1.40-2.61)	<0.001	1.80	(1.36-2.40)	<0.001	1.76	(1.31-2.36)	<0.001

CVD refers to participants with self-reported myocardial infarction, coronary artery bypass grafting, percutaneous coronary intervention, stroke, or peripheral arterial disease intervention.
Reference category for CCTA stenosis: no stenosis.
All models refer to the extended adjusted model: adjusted for age at conscription, age at SCAPIS, site in conscription, site in SCAPIS, conscription year, BMI at conscription (linear and quadratic terms), smoking at conscription, and education level at SCAPIS.
Normal BP: SBP <120 and DBP <80 mmHg; elevated BP: SBP =120-129 and DBP <80 mmHg; hypertension stage 1: SBP =130-139 or DBP =80-89 mmHg; hypertension stage 2: SBP ≥140 or DBP ≥90 mmHg.
ACC/AHA: American College of Cardiology/American Heart Association, BMI: body mass index, BP: blood pressure, CI: confidence interval, CVD: cardiovascular disease, OR: odds ratio, ref.: reference, SCAPIS: Swedish CArdioPulmonary bioImage Study.

© 2025 Herraiz-Adillo Á et al. *JAMA Cardiology*.

eTable 13. Sensitivity Analysis, Associations of 2025 ACC/AHA Blood Pressure Categories in Adolescence With Coronary Stenosis in Middle Age Considering Antihypertensive Medication in Middle Age[2]

	Considering the 11 relevant coronary segments (Ref. analysis) n = 9110			Participants not taking antihypertensives at SCAPIS n = 6849			Participants taking antihypertensives at SCAPIS n = 2261		
	**OR**	**(95% CI)**	**P**	**OR**	**(95% CI)**	**P**	**OR**	**(95% CI)**	**P**
**2025 ACC/AHA BP classification**									
**Coronary stenosis**									
**1-49%**									
**Normal BP, ref.**	1.00	-	-	1.00	-	-	1.00	-	-
**Elevated BP**	1.02	(0.90-1.17)	0.73	1.02	(0.88-1.18)	0.76	1.10	(0.80-1.51)	0.56
**Hypertension stage 1**	1.06	(0.93-1.20)	0.40	1.01	(0.87-1.16)	0.93	0.96	(0.71-1.31)	0.81
**Hypertension stage 2**	1.36	(1.16-1.58)	<0.001	1.27	(1.07-1.52)	0.008	0.82	(0.58-1.15)	0.25
**≥ 50%**									
**Normal BP, ref.**	1.00	-	-	1.00	-	-	1.00	-	-
**Elevated BP**	1.31	(1.02-1.68)	0.03	1.30	(0.95-1.79)	0.11	1.21	(0.80-1.80)	0.36
**Hypertension stage 1**	1.35	(1.06-1.72)	0.01	1.27	(0.93-1.75)	0.13	1.09	(0.75-1.60)	0.65
**Hypertension stage 2**	1.84	(1.40-2.42)	<0.001	1.83	(1.27-2.64)	0.001	0.95	(0.62-1.44)	0.81

Antihypertensive medication at SCAPIS combines information from the Prescribed Drugs Register with self-reported information from the SCAPIS core questionnaire.
Reference category for CCTA stenosis: no stenosis.
All models refer to the extended adjusted model: adjusted for age at conscription, age at SCAPIS, site in conscription, site in SCAPIS, conscription year, BMI at conscription (linear and quadratic terms), smoking at conscription, and education level at SCAPIS.
Normal BP: SBP <120 and DBP <80 mmHg; elevated BP: SBP =120-129 and DBP <80 mmHg; hypertension stage 1: SBP =130-139 or DBP =80-89 mmHg; hypertension stage 2: SBP ≥140 or DBP ≥90 mmHg.
ACC/AHA: American College of Cardiology/American Heart Association, BMI: body mass index, BP: blood pressure, CI: confidence interval, OR: odds ratio, ref.: reference, SCAPIS: Swedish CArdioPulmonary bioImage Study.

© 2025 Herraiz-Adillo Á et al. *JAMA Cardiology*.

eTable 14. Multinomial Logistic Regression, Odds Ratios for the Association of 2025 ACC/AHA Blood Pressure Categories in Adolescence With Coronary Artery Calcium and Carotid Atherosclerosis in Middle Age[2]

	Basic adjustment			Extended adjustment		
**2025 ACC/AHA BP classification**	**OR**	**(95% CI)**	**P**	**OR**	**(95% CI)**	**P**
**CAC score**						
**1-99**						
**Normal BP, ref.**	1.00	-	-	1.00	-	-
**Elevated BP**	1.03	(0.90-1.18)	0.65	1.02	(0.88-1.17)	0.82
**Hypertension stage 1**	1.05	(0.91-1.20)	0.51	1.02	(0.89-1.17)	0.82
**Hypertension stage 2**	1.40	(1.19-1.64)	<0.001	1.34	(1.14-1.58)	<0.001
**≥100**						
**Normal BP, ref.**	1.00	-	-	1.00	-	-
**Elevated BP**	1.30	(1.08-1.57)	0.01	1.24	(1.03-1.50)	0.02
**Hypertension stage 1**	1.41	(1.18-1.69)	<0.001	1.31	(1.09-1.58)	0.01
**Hypertension stage 2**	1.90	(1.55-2.34)	<0.001	1.73	(1.40-2.13)	<0.001
**Ultrasound carotid plaque**						
**Unilateral**						
**Normal BP, ref.**	1.00	-	-	1.00	-	-
**Elevated BP**	0.97	(0.84-1.11)	0.66	0.97	(0.85-1.12)	0.70
**Hypertension stage 1**	1.13	(0.99-1.29)	0.08	1.14	(1.00-1.31)	0.06
**Hypertension stage 2**	1.14	(0.97-1.33)	0.12	1.16	(0.98-1.36)	0.08
**Bilateral**						
**Normal BP, ref.**	1.00	-	-	1.00	-	-
**Elevated BP**	1.13	(0.98-1.31)	0.10	1.15	(1.00-1.33)	0.06
**Hypertension stage 1**	1.26	(1.10-1.45)	0.001	1.31	(1.13-1.51)	<0.001
**Hypertension stage 2**	1.43	(1.21-1.68)	<0.001	1.51	(1.27-1.78)	<0.001

Reference category for CAC score: 0 Agatston units. Reference category for carotid plaques: no plaque.
Basic adjusted model: adjusted for age at conscription, age at SCAPIS, site in conscription, site in SCAPIS, and conscription year.
Extended adjusted model: basic adjustment plus BMI at conscription (linear and quadratic terms), smoking at conscription, and education level at SCAPIS.
Normal BP: SBP <120 and DBP <80 mmHg; elevated BP: SBP =120-129 and DBP <80 mmHg; hypertension stage 1: SBP =130-139 or DBP =80-89 mmHg; hypertension stage 2: SBP ≥140 or DBP ≥90 mmHg.
ACC/AHA: American College of Cardiology/American Heart Association, BMI: body mass index, BP: blood pressure, CAC: coronary artery calcium, CI: confidence interval, OR: odds ratio, ref.: reference, SCAPIS: Swedish CArdioPulmonary bioImage Study.

© 2025 Herraiz-Adillo Á et al. *JAMA Cardiology*.

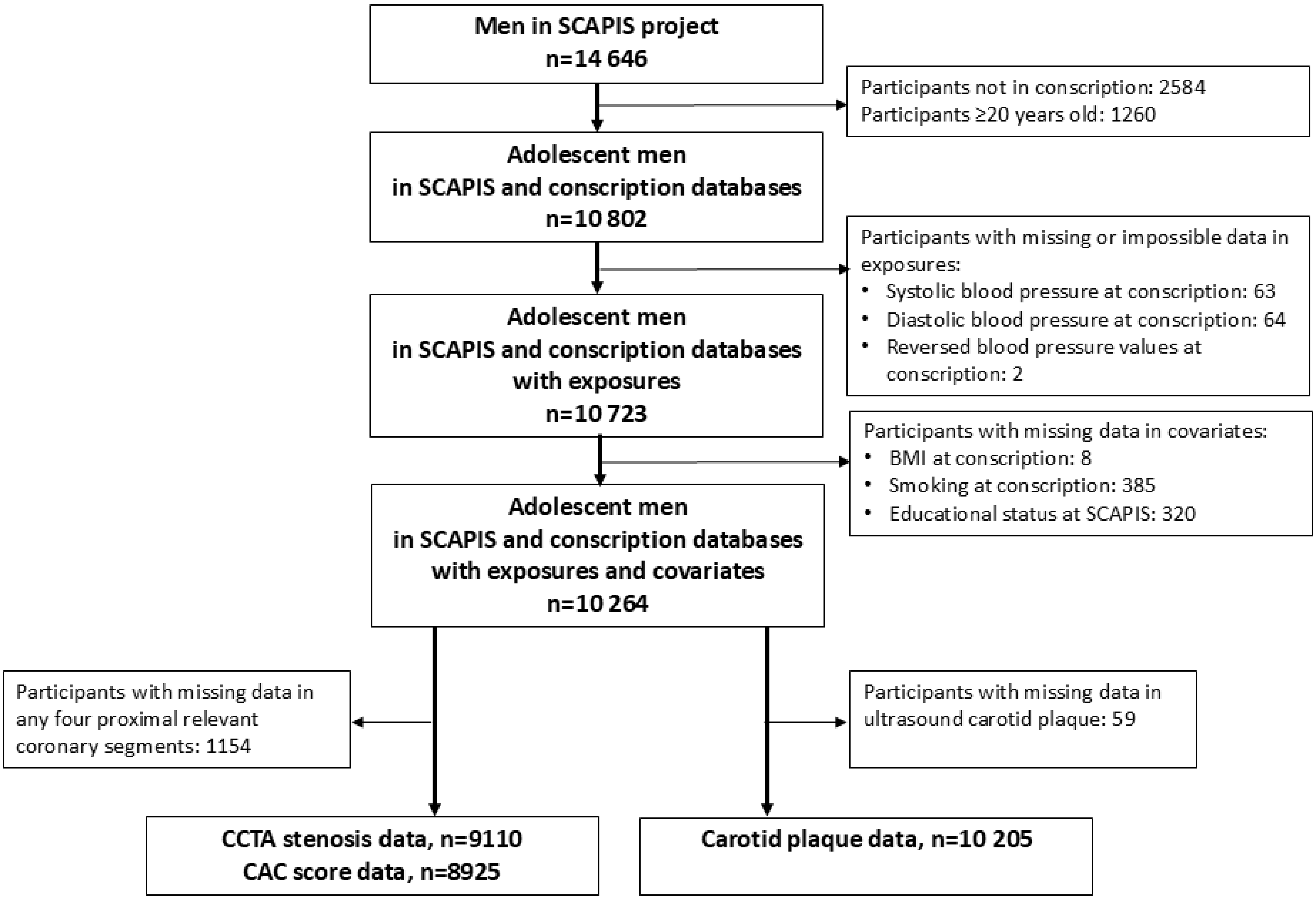

eFigure 1. Flowchart of the Study

BMI: body mass index, CAC: coronary artery calcium, CCTA: coronary computed tomography angiography, SCAPIS: Swedish CArdioPulmonary bioImage Study.

© 2025 Herraiz-Adillo Á et al. *JAMA Cardiology*.

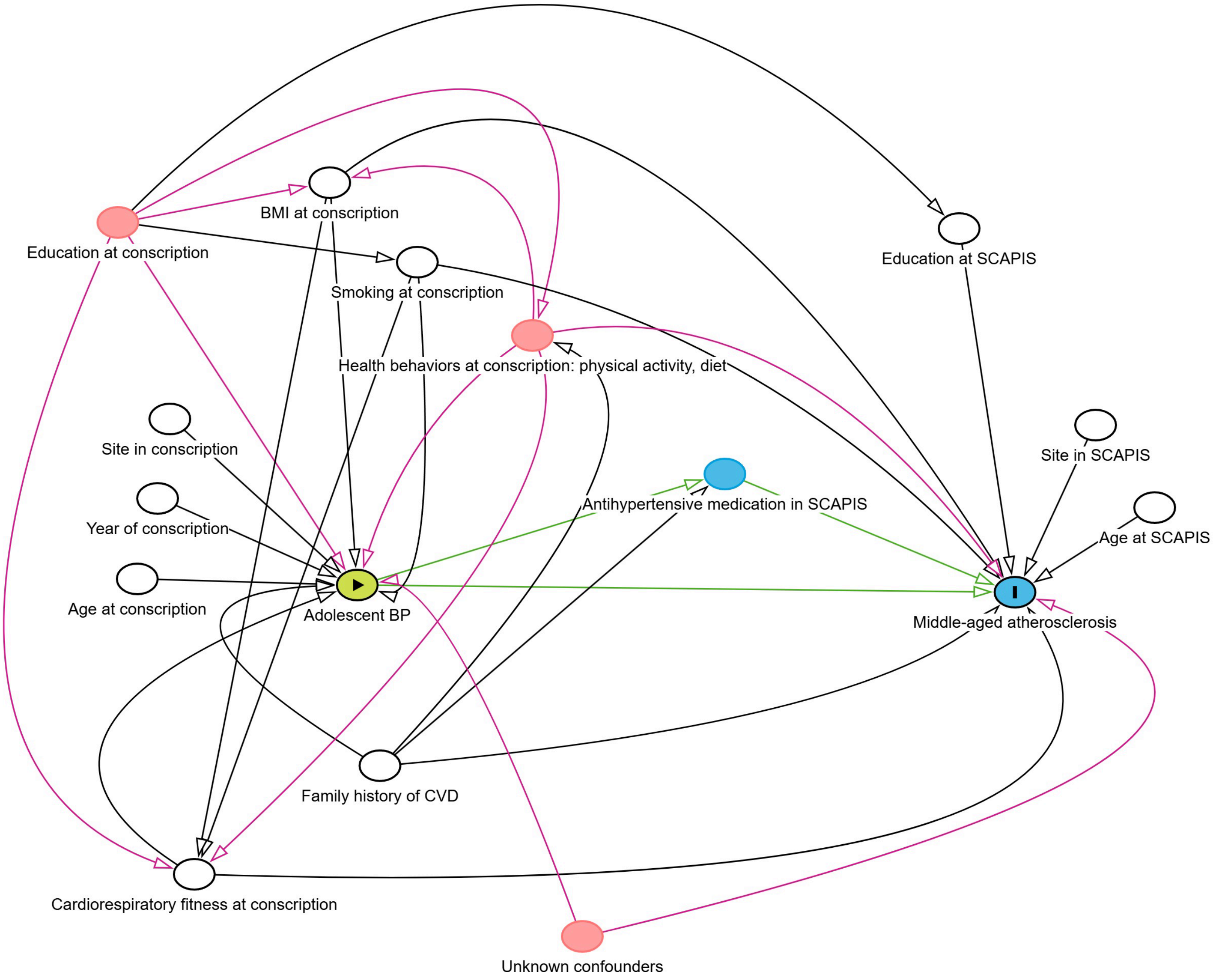

eFigure 2. Directed Acyclic Graph for the Association of Blood Pressure in Adolescence With Atherosclerosis in Middle Age[15]

The graph was created with DAGitty (https://dagitty.net)

BMI: body mass index, BP: blood pressure, SCAPIS: Swedish CArdioPulmonary bioImage Study.

Exposure, Outcome, Unadjusted variable, Adjusted variable, Causal path, Biasing path.

© 2025 Herraiz-Adillo Á et al. *JAMA Cardiology*.

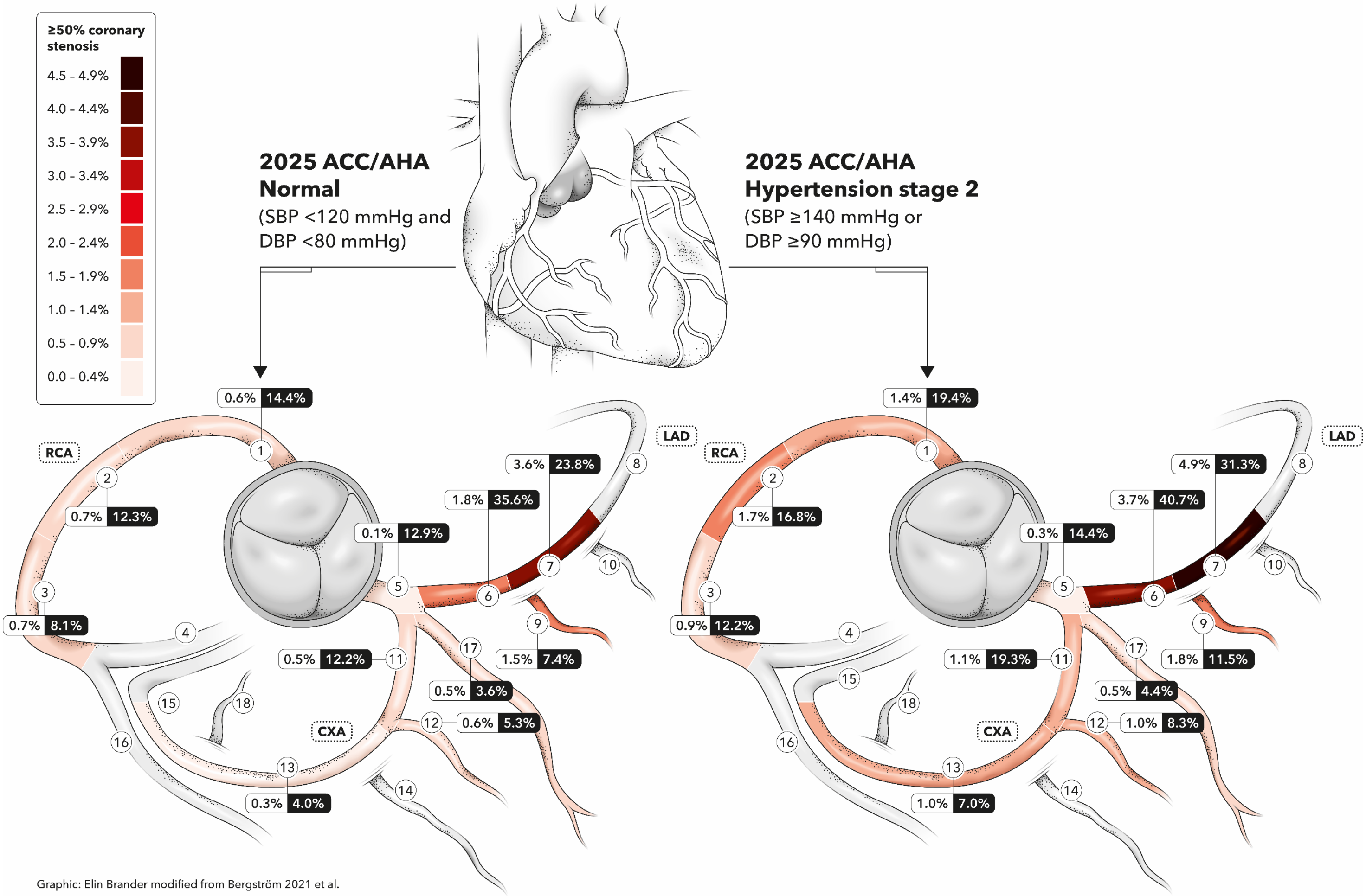

eFigure 3. Adjusted Prevalences for the Association of 2025 ACC/AHA Blood Pressure Categories in Adolescence With Coronary Stenosis in Middle Age in the 11 Most Relevant Coronary Segments

Heatmap and white squares depict the adjusted (by age at conscription, site in conscription, year of conscription, BMI at conscription, smoking duration at conscription, age at SCAPIS, site in SCAPIS and educational level at SCAPIS) prevalence for the association of 2025 ACC/AHA blood pressure categories (normal versus hypertension stage 2) and ≥50% coronary stenosis, while black squares depict the adjusted prevalences for 1-49% coronary stenosis.

ACC/AHA: American College of Cardiology/American Heart Association, BMI: body mass index, CXA: circumflex artery, DBP: diastolic blood pressure, LAD: left anterior descending artery, RCA: right coronary artery, SBP: systolic blood pressure, SCAPIS: Swedish CArdioPulmonary bioImage Study. Adapted from Circulation[7] and J Cardiovasc Comput Tomogr[8], with permission.

© 2025 Herraiz-Adillo Á et al. *JAMA Cardiology*.

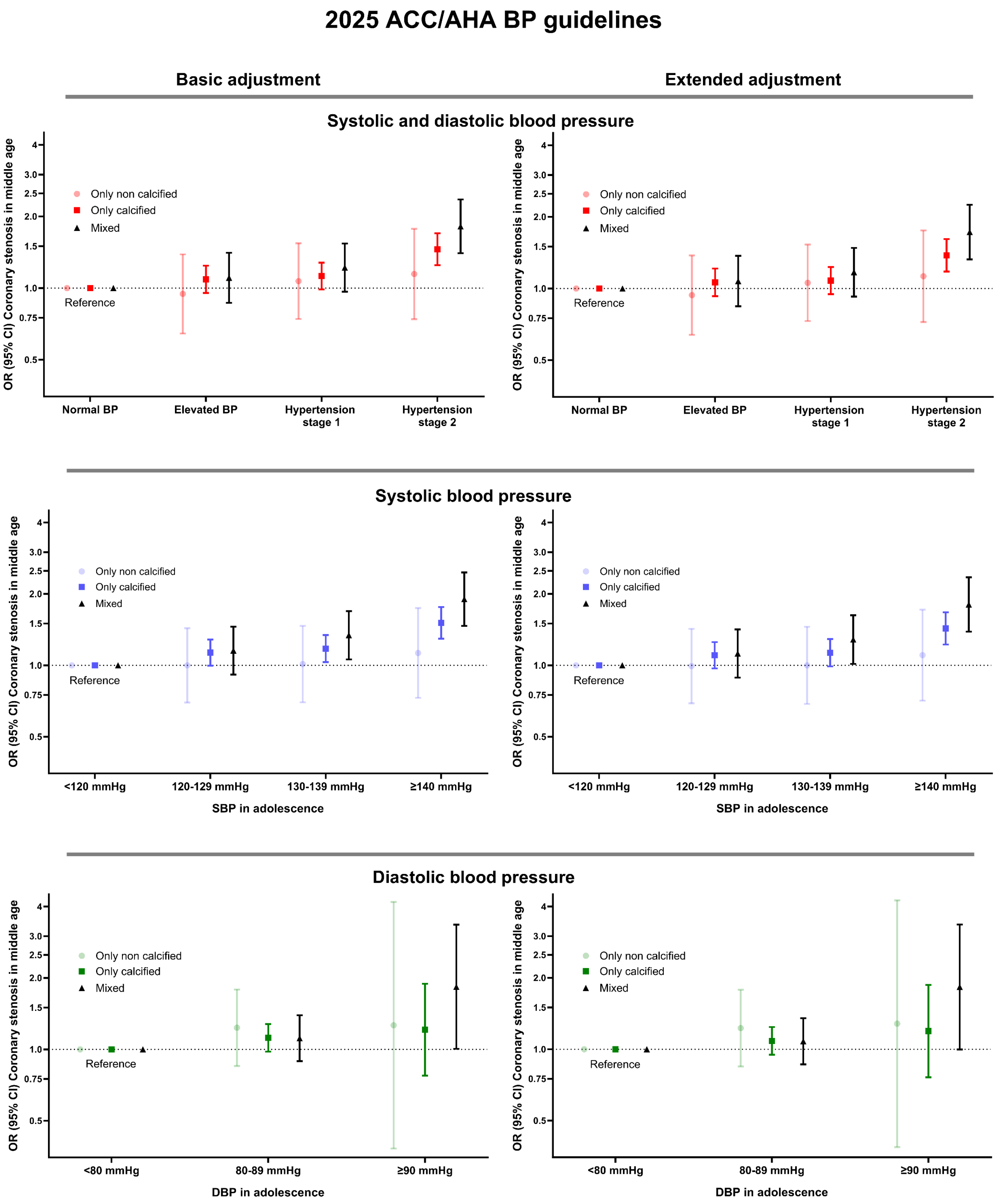

eFigure 4. Associations of 2025 ACC/AHA Blood Pressure Categories in Adolescence With Composition of the Coronary Plaques in Middle Age[2]

Composition is categorized as: no plaque, only non-calcified plaque/s (all identified plaque/s are non-calcified), only calcified plaque/s (all identified plaque/s are calcified), and mixed composition (presence of both calcified and non-calcified segments in the arterial tree). Basic adjusted models are adjusted for age at conscription, site in conscription, year of conscription, age at SCAPIS and site in SCAPIS. Extended adjusted models are adjusted for age at conscription, site in conscription, year of conscription, BMI at conscription, smoking duration at conscription, age at SCAPIS, site in SCAPIS and educational level at SCAPIS.

© 2025 Herraiz-Adillo Á et al. *JAMA Cardiology*.

ACC/AHA: American College of Cardiology/American Heart Association, BMI: body mass index, BP: blood pressure, CI: confidence interval, DBP: diastolic blood pressure, OR: odds ratio, SBP: systolic blood pressure, SCAPIS: Swedish CArdioPulmonary bioImage Study.

© 2025 Herraiz-Adillo Á et al. *JAMA Cardiology*.

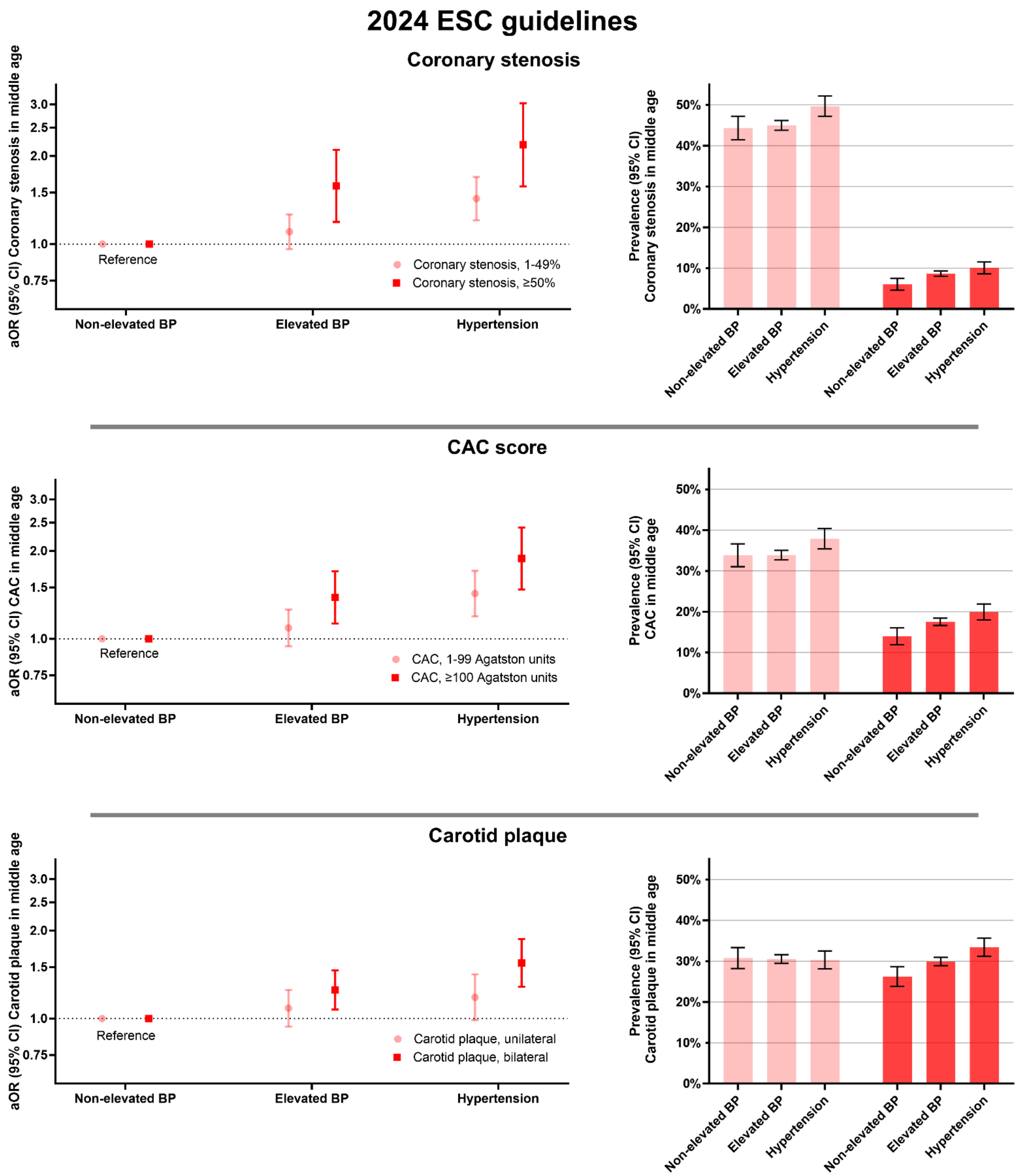

eFigure 5. Associations of 2024 ESC Blood Pressure Categories in Adolescence With Coronary and Carotid Atherosclerosis in Middle Age[3]

Multinomial logistic models depicting aORs and prevalences are adjusted for age at conscription, site in conscription, year of conscription, BMI at conscription, smoking duration at conscription, age at SCAPIS, site in SCAPIS and educational level at SCAPIS.

Non-elevated BP: SBP<120 and DBP<70 mmHg; elevated BP: SBP = 120-139 or DBP 70-89 mmHg; hypertension: SBP≥140 or DBP≥90 mmHg.

aOR: adjusted odds ratio, BMI: body mass index, BP: blood pressure, CAC: coronary artery calcium, CI: confidence interval, ESC: European Society of Cardiology, SCAPIS: Swedish CArdioPulmonary bioImage Study.

© 2025 Herraiz-Adillo Á et al. *JAMA Cardiology*.

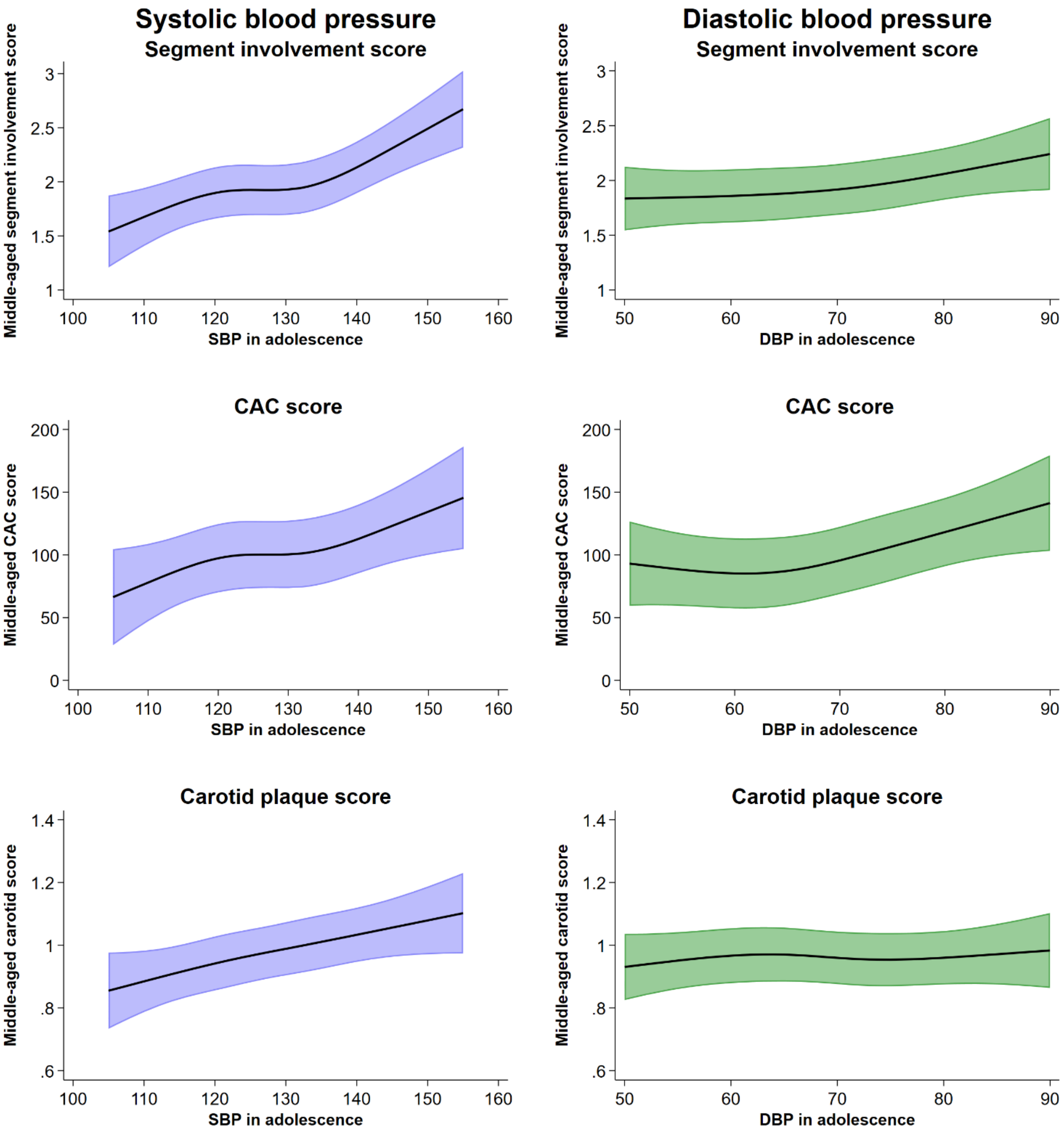

eFigure 6. Associations Across Restricted Cubic Splines of Systolic and Diastolic Blood Pressures in Adolescence With Coronary (Segment Involvement Score and CAC Score) and Carotid (Carotid Plaque Score) Atherosclerosis in Middle Age

Linear models are adjusted for age at conscription, site in conscription, year of conscription, BMI at conscription, smoking duration at conscription, age at SCAPIS, site in SCAPIS and educational level at SCAPIS. X-axes are trimmed to depict the associations for the 1st to 99th percentile of systolic and diastolic blood pressure values.

BMI: body mass index, CAC: coronary artery calcium, DBP: diastolic blood pressure, SBP: systolic blood pressure, SCAPIS: Swedish CArdioPulmonary bioImage Study.

© 2025 Herraiz-Adillo Á et al. *JAMA Cardiology*.

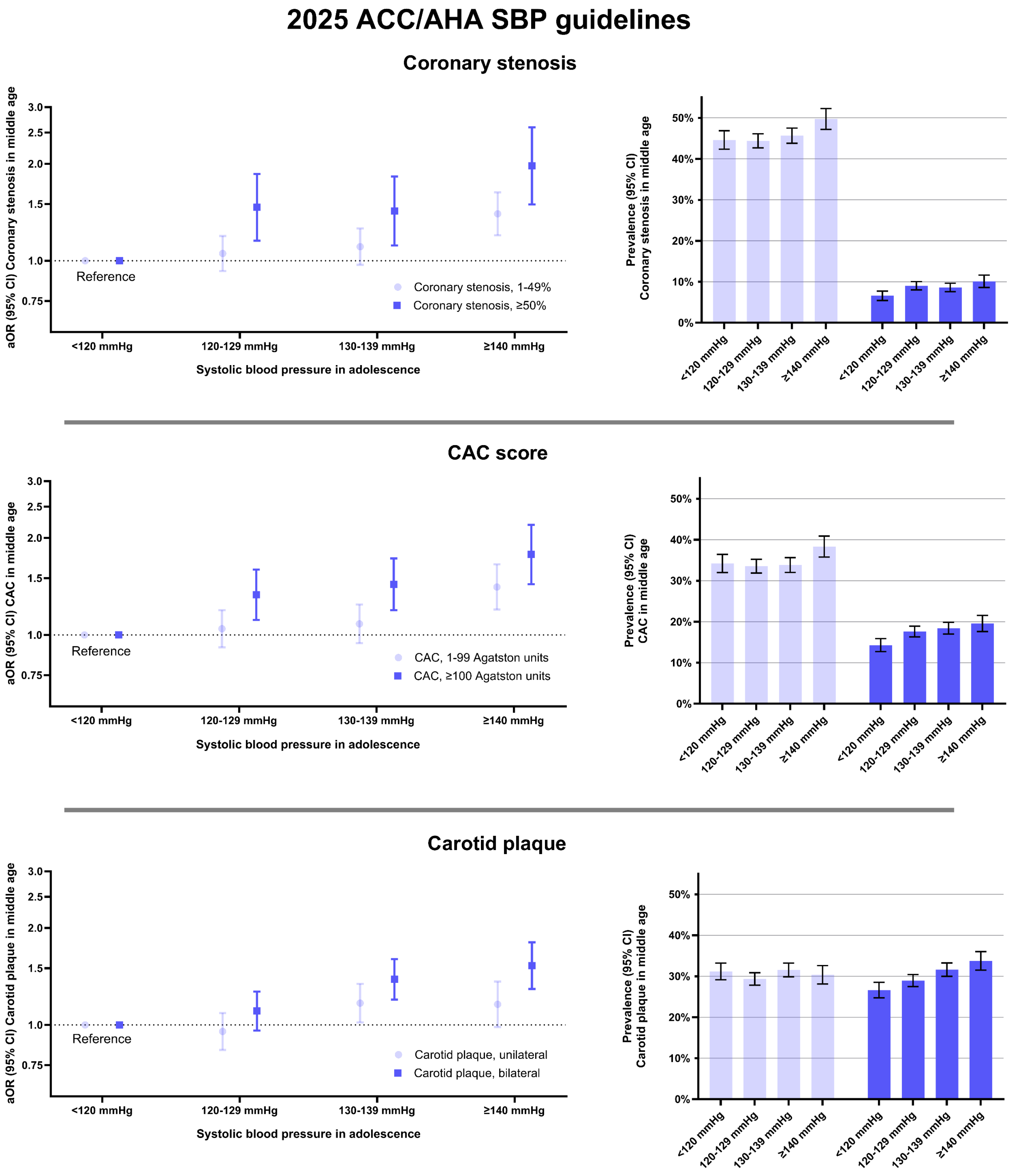

eFigure 7. Associations of 2025 ACC/AHA Systolic Blood Pressure Categories in Adolescence With Coronary and Carotid Atherosclerosis in Middle Age[2]

Multinomial logistic models depicting aORs and prevalences are adjusted for age at conscription, site in conscription, year of conscription, BMI at conscription, smoking duration at conscription, age at SCAPIS, site in SCAPIS and educational level at SCAPIS. ACC/AHA: American College of Cardiology/American Heart Association, aOR: adjusted odds ratio, BMI: body mass index, CAC: coronary artery calcium, CI: confidence interval, SBP: systolic blood pressure, SCAPIS: Swedish CArdioPulmonary bioImage Study.

© 2025 Herraiz-Adillo Á et al. *JAMA Cardiology*.

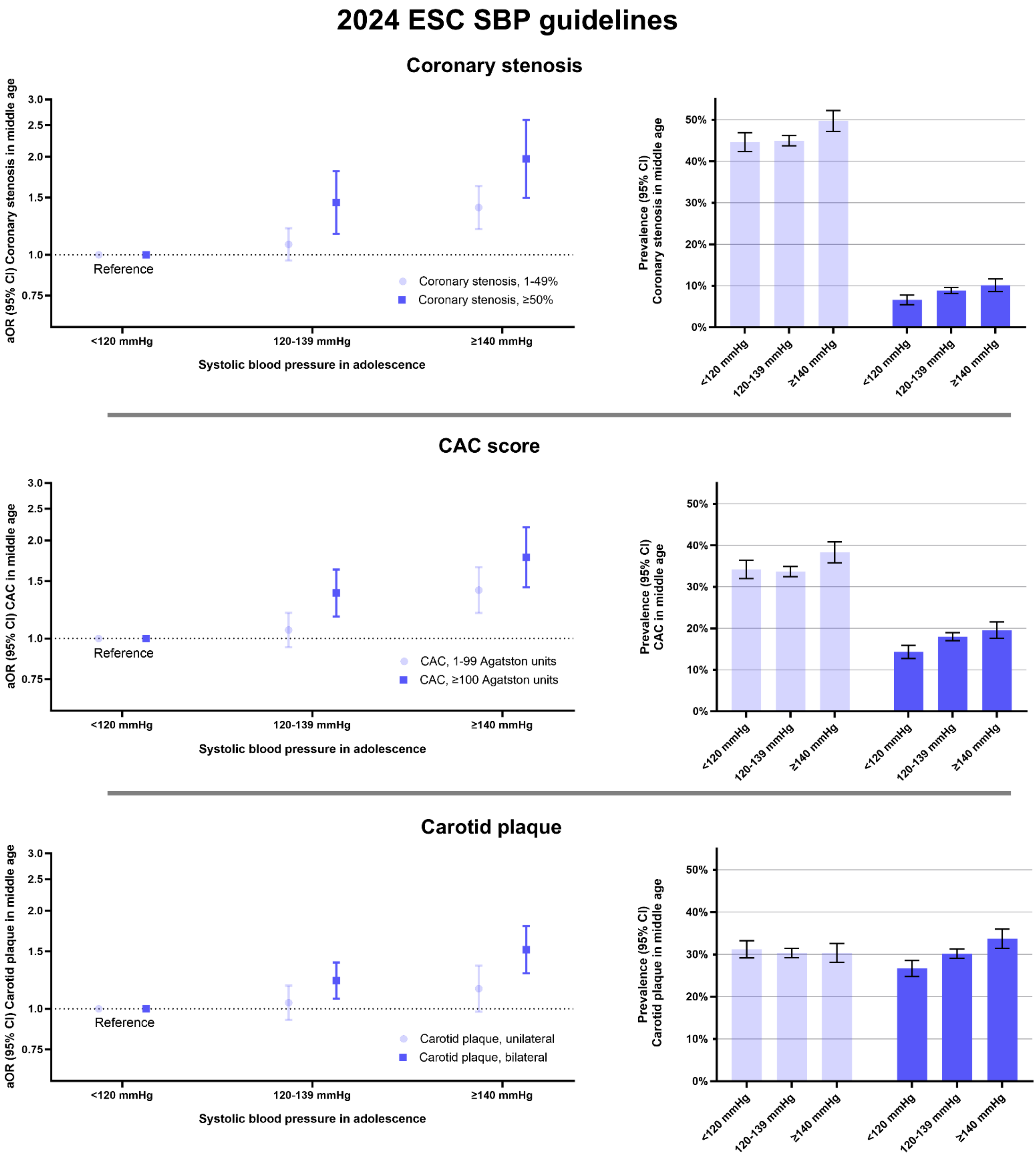

eFigure 8. Associations of 2024 ESC Systolic Blood Pressure Categories in Adolescence With Coronary and Carotid Atherosclerosis in Middle Age[3]

Multinomial logistic models depicting aORs and prevalences are adjusted for age at conscription, site in conscription, year of conscription, BMI at conscription, smoking duration at conscription, age at SCAPIS, site in SCAPIS and educational level at SCAPIS.

aOR: adjusted odds ratio, BMI: body mass index, CAC: coronary artery calcium, CI: confidence interval, ESC: European Society of Cardiology, SBP: systolic blood pressure, SCAPIS: Swedish CArdioPulmonary bioImage Study.

© 2025 Herraiz-Adillo Á et al. *JAMA Cardiology*.

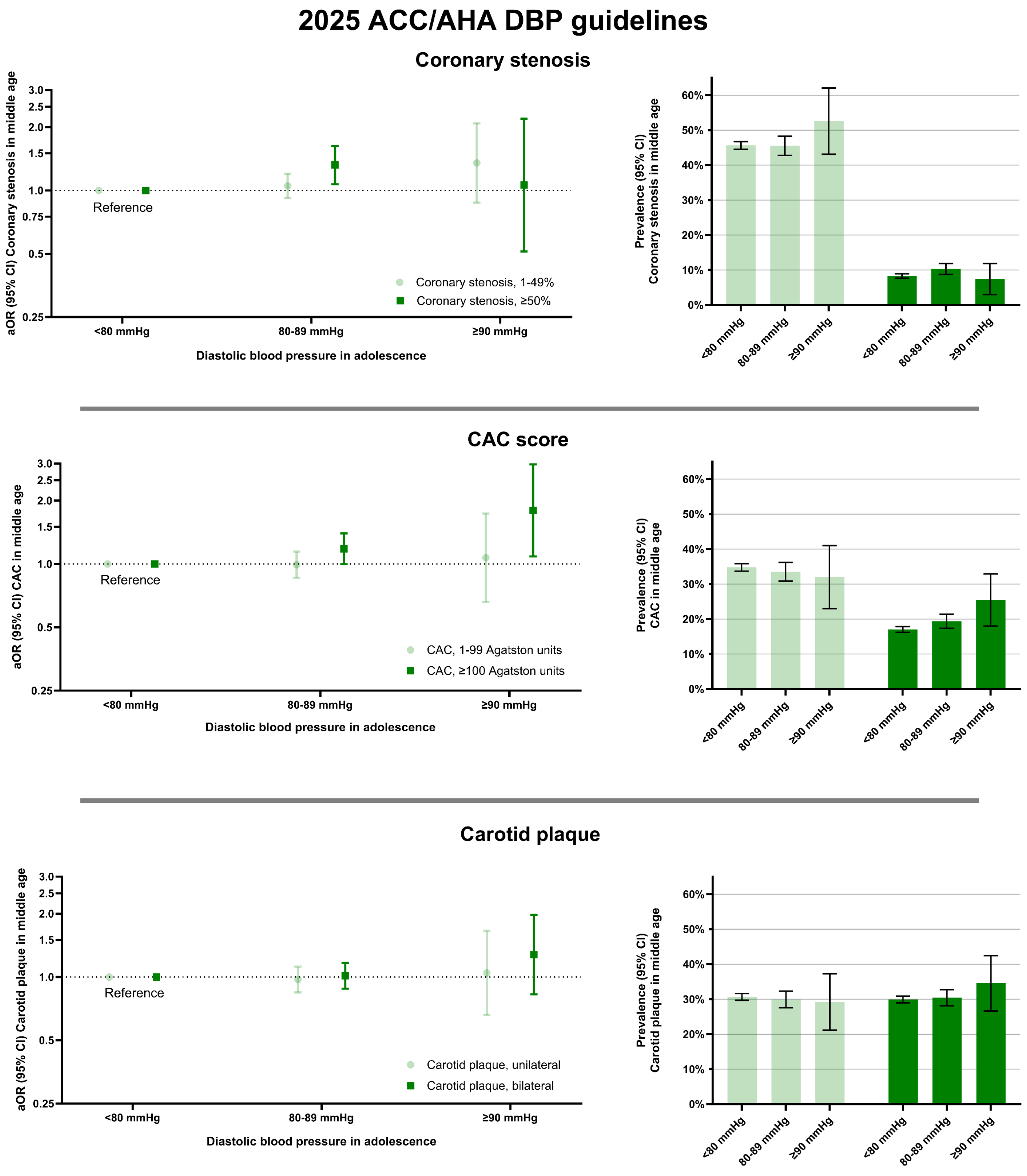

eFigure 9. Associations of 2025 ACC/AHA Diastolic Blood Pressure Categories in Adolescence With Coronary and Carotid Atherosclerosis in Middle Age[2]

Multinomial logistic models depicting aORs and prevalences are adjusted for age at conscription, site in conscription, year of conscription, BMI at conscription, smoking duration at conscription, age at SCAPIS, site in SCAPIS and educational level at SCAPIS.

ACC/AHA: American College of Cardiology/American Heart Association, aOR: adjusted odds ratio, BMI: body mass index, CAC: coronary artery calcium, CI: confidence interval, DBP: diastolic blood pressure, SCAPIS: Swedish CArdioPulmonary bioImage Study.

© 2025 Herraiz-Adillo Á et al. *JAMA Cardiology*.

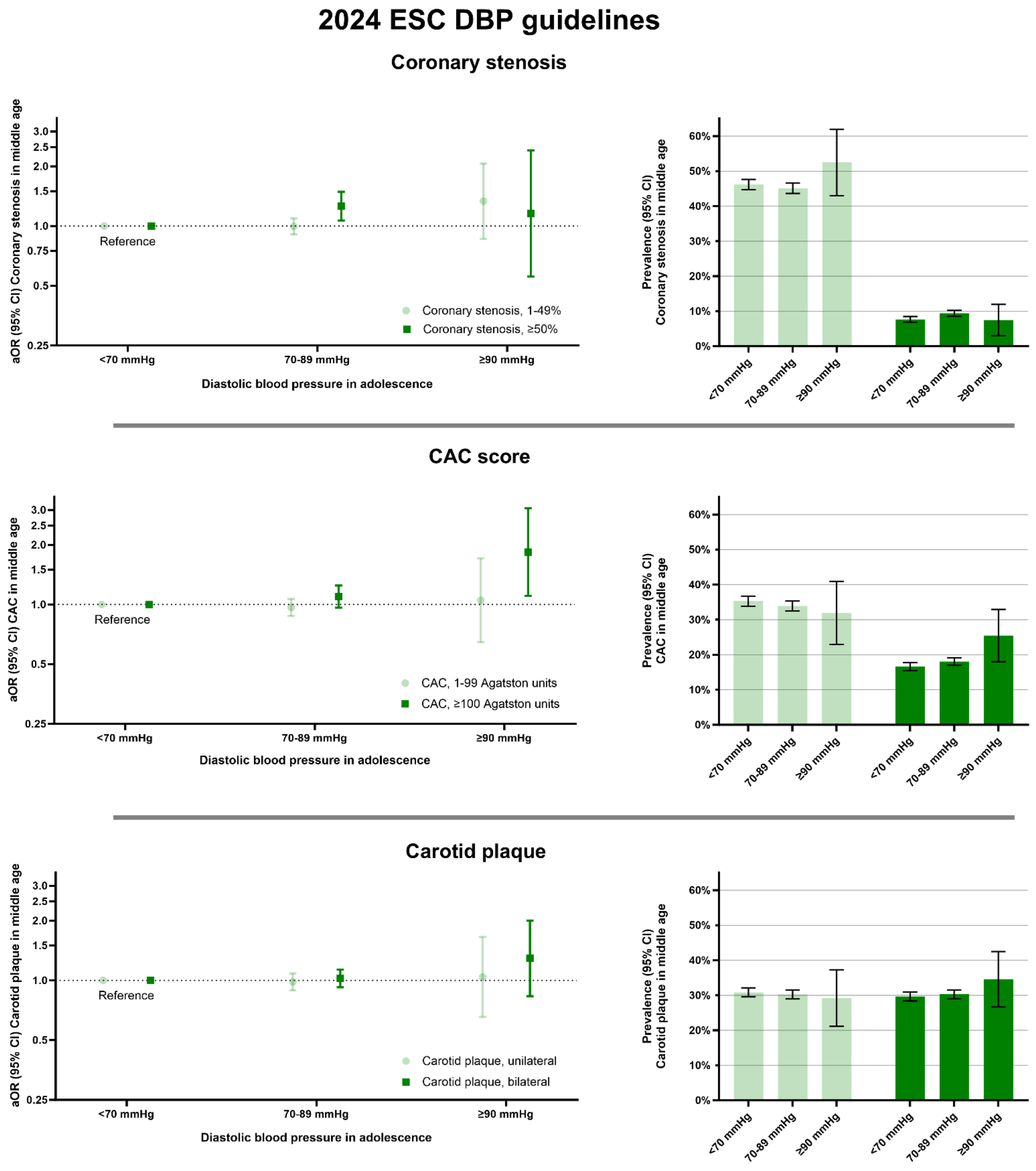

eFigure 10. Associations of 2024 ESC Diastolic Blood Pressure Categories in Adolescence With Coronary and Carotid Atherosclerosis in Middle Age[3]

Multinomial logistic models depicting aORs and prevalences are adjusted for age at conscription, site in conscription, year of conscription, BMI at conscription, smoking duration at conscription, age at SCAPIS, site in SCAPIS and educational level at SCAPIS.

aOR: adjusted odds ratio, BMI: body mass index, CAC: coronary artery calcium, CI: confidence interval, DBP: diastolic blood pressure, ESC: European Society of Cardiology, SCAPIS: Swedish CArdioPulmonary bioImage Study.

© 2025 Herraiz-Adillo Á et al. *JAMA Cardiology*.

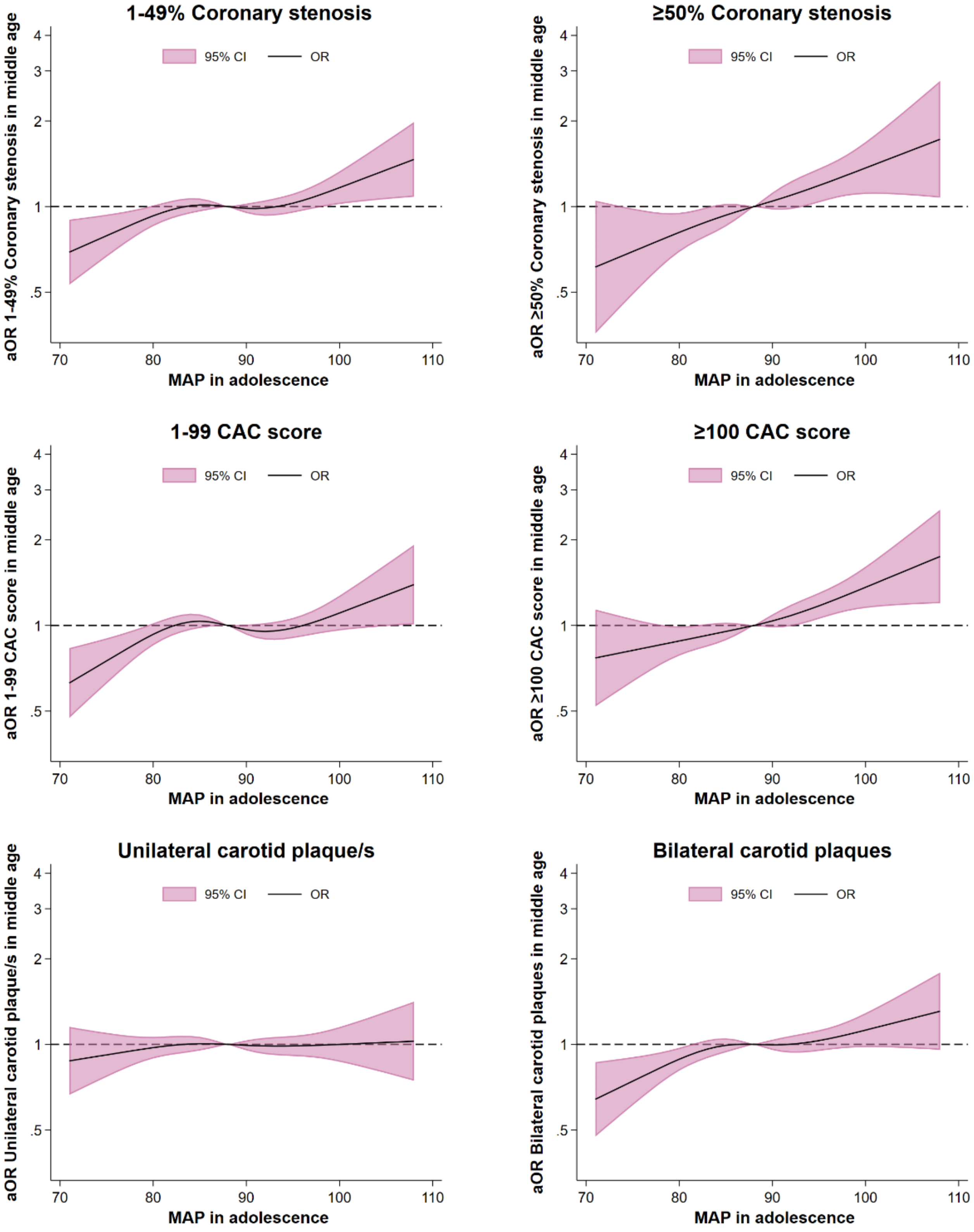

eFigure 11. Associations Across Restricted Cubic Splines of Mean Arterial Pressure in Adolescence With Coronary and Carotid Atherosclerosis in Middle Age

Multinomial logistic models are adjusted for age at conscription, site in conscription, year of conscription, BMI at conscription, smoking duration at conscription, age at SCAPIS, site in SCAPIS and educational level at SCAPIS. X-axes are trimmed to depict the associations for the 1st to 99th percentile of MAP values. Reference is set at the median of MAP (88 mmHg).

aOR: adjusted odds ratio, BMI: body mass index, CAC: coronary artery calcium, CI: confidence interval, MAP: mean arterial pressure, SCAPIS: Swedish CArdioPulmonary bioImage Study.

© 2025 Herraiz-Adillo Á et al. *JAMA Cardiology*.

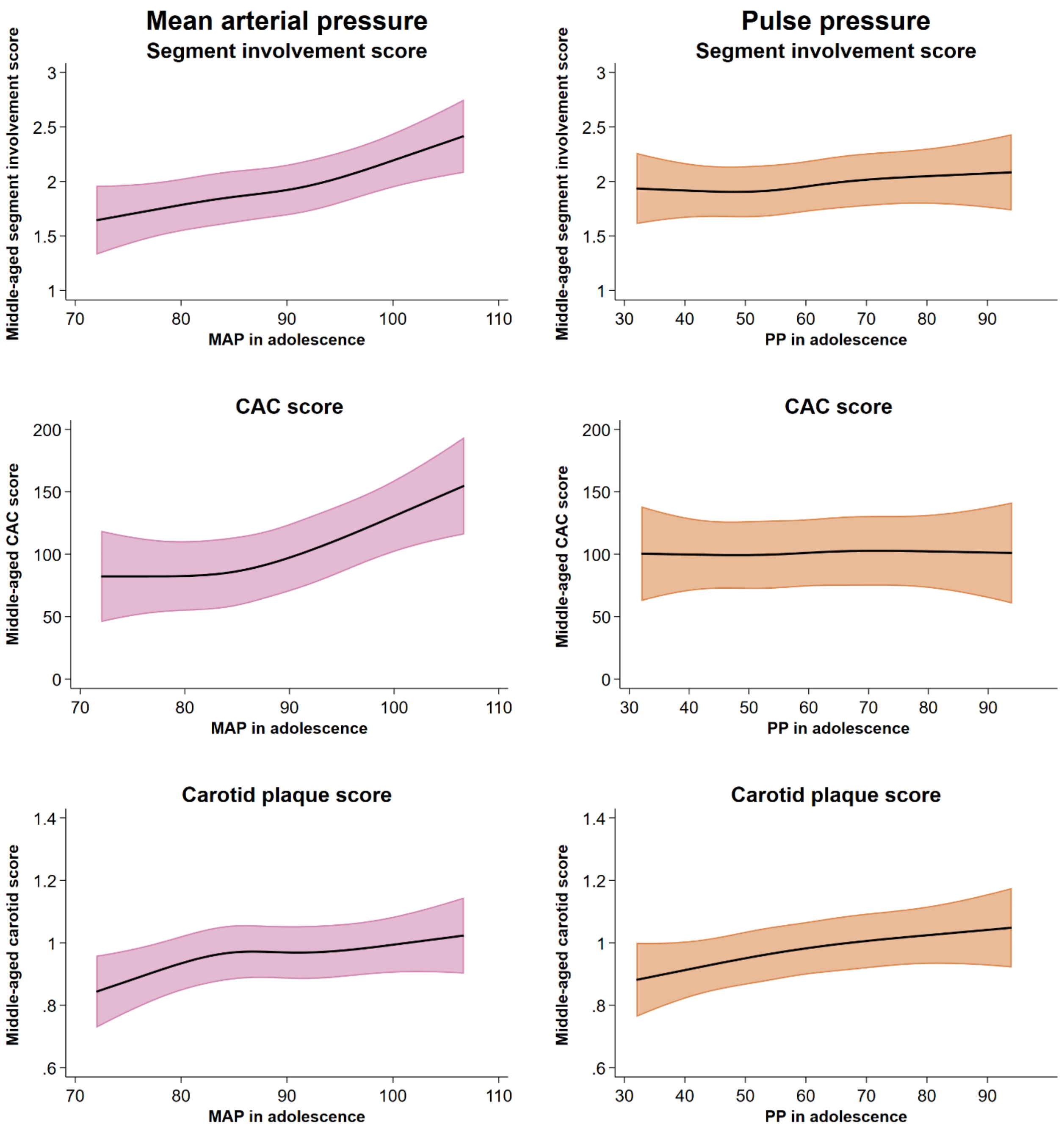

eFigure 12. Associations Across Restricted Cubic Splines of Mean Arterial Pressure and Pulse Pressure in Adolescence With Coronary (Segment Involvement Score and CAC Score) and Carotid (Carotid Plaque Score) Atherosclerosis in Middle Age

Linear models are adjusted for age at conscription, site in conscription, year of conscription, BMI at conscription, smoking duration at conscription, age at SCAPIS, site in SCAPIS and educational level at SCAPIS. X-axes are trimmed to depict the associations for the 1st to 99th percentile of mean arterial pressure and pulse pressure values.

BMI: body mass index, CAC: coronary artery calcium, MAP: mean arterial pressure, PP: pulse pressure, SCAPIS: Swedish CArdioPulmonary bioImage Study.

© 2025 Herraiz-Adillo Á et al. *JAMA Cardiology*.

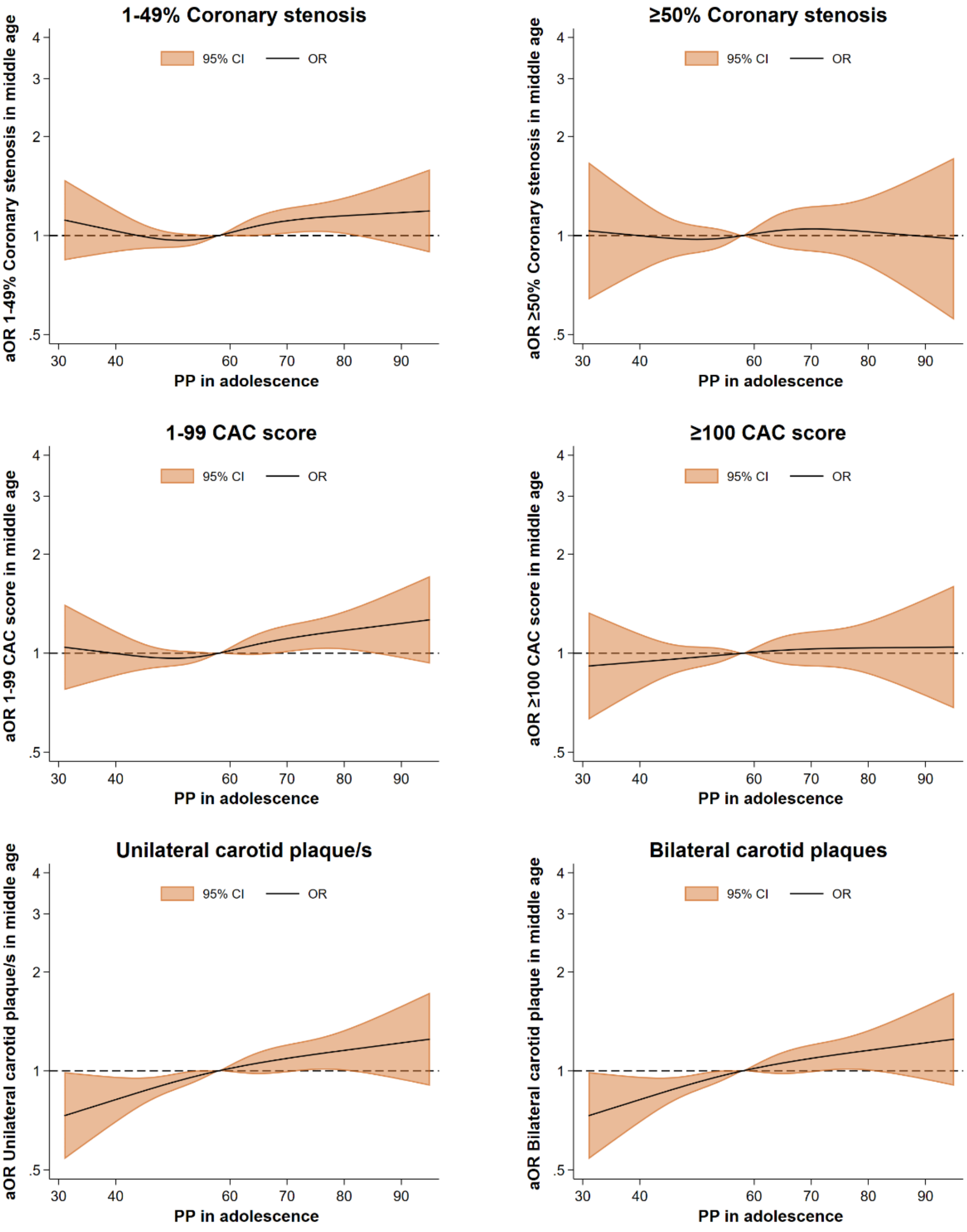

eFigure 13. Associations Across Restricted Cubic Splines of Pulse Pressure in Adolescence With Coronary and Carotid Atherosclerosis in Middle Age

Multinomial logistic models are adjusted for age at conscription, site in conscription, year of conscription, BMI at conscription, smoking duration at conscription, age at SCAPIS, site in SCAPIS and educational level at SCAPIS. X-axes are trimmed to depict the associations for the 1st to 99th percentile of PP values. Reference is set at the median of PP (58 mmHg).

aOR: adjusted odds ratio, BMI: body mass index, CAC: coronary artery calcium, CI: confidence interval, PP: pulse pressure, SCAPIS: Swedish CArdioPulmonary bioImage Study.

© 2025 Herraiz-Adillo Á et al. *JAMA Cardiology*.

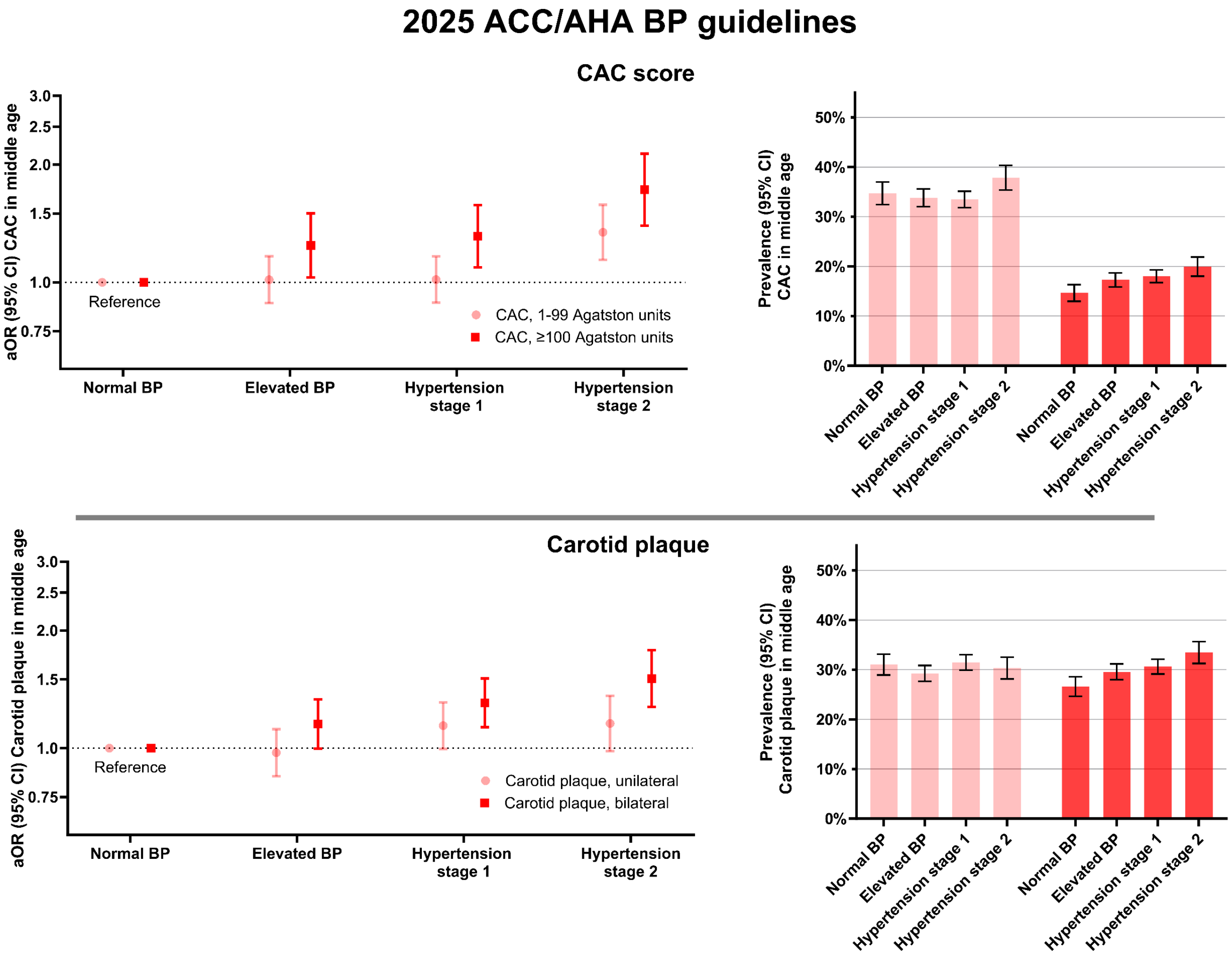

eFigure 14. Associations of 2025 ACC/AHA Blood Pressure Categories in Adolescence With CAC Score and Carotid Plaque in Middle Age[2]

Multinomial logistic models depicting aORs and prevalences are adjusted for age at conscription, site in conscription, year of conscription, BMI at conscription, smoking duration at conscription, age at SCAPIS, site in SCAPIS and educational level at SCAPIS.

Normal BP: SBP <120 and DBP <80 mmHg; elevated BP: SBP =120-129 and DBP <80 mmHg; hypertension stage 1: SBP =130-139 or DBP =80-89 mmHg; hypertension stage 2: SBP ≥140 or DBP ≥90 mmHg.

ACC/AHA: American College of Cardiology/American Heart Association, aOR: adjusted odds ratio, BMI: body mass index, BP: blood pressure, CI: confidence interval, CT: Computed Tomography, SCAPIS: Swedish CArdioPulmonary bioImage Study.

© 2025 Herraiz-Adillo Á et al. *JAMA Cardiology*.

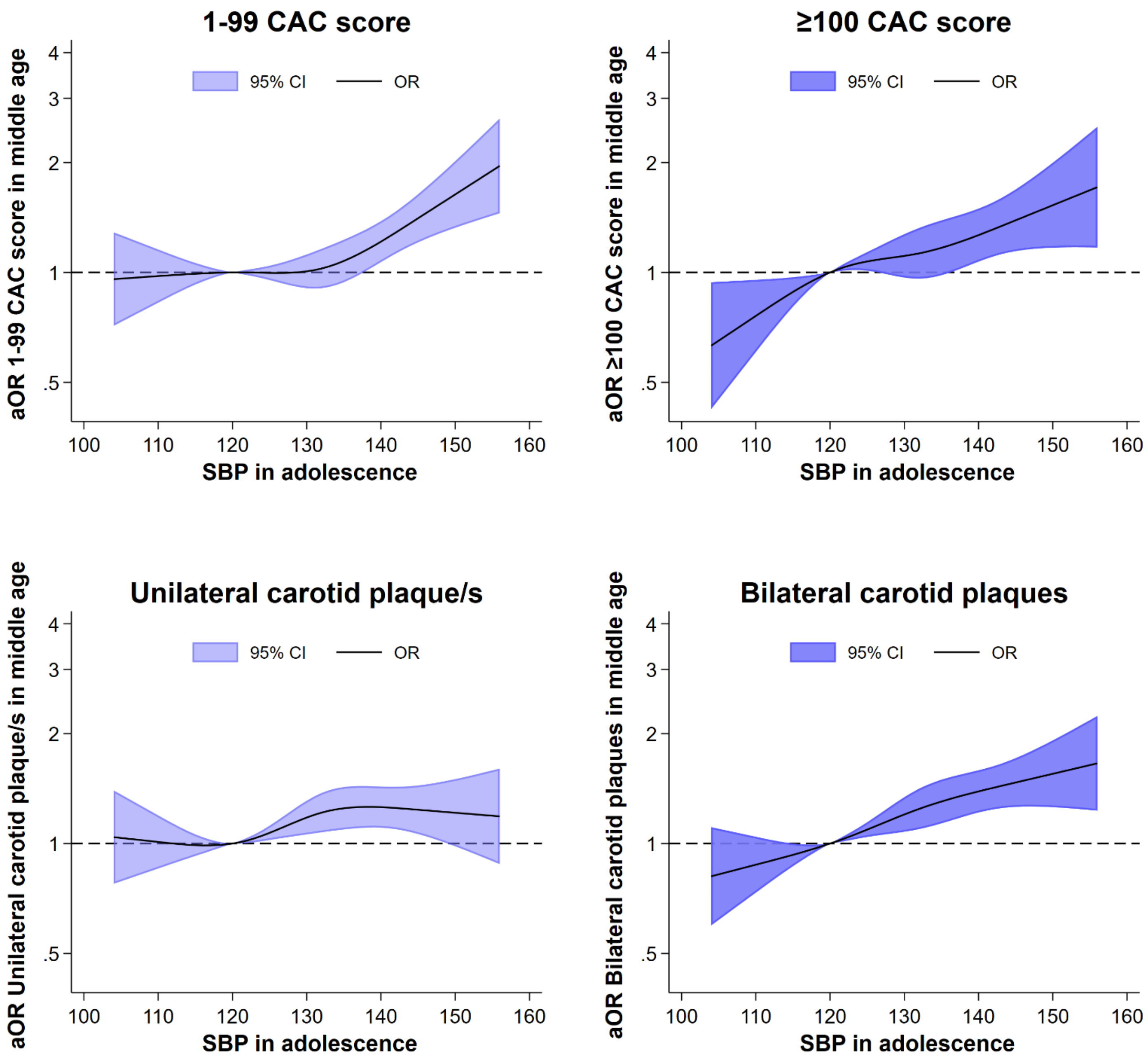

eFigure 15. Associations Across Restricted Cubic Splines of Systolic Blood Pressure in Adolescence With CAC Score and Carotid Plaques in Middle Age

Multinomial logistic models are adjusted for age at conscription, site in conscription, year of conscription, BMI at conscription, smoking duration at conscription, age at SCAPIS, site in SCAPIS and educational level at SCAPIS. X-axes are trimmed to depict the associations for the 1st to 99th percentile of diastolic blood pressure values. Reference is set at 120 mmHg.

aOR: adjusted odds ratio, BMI: body mass index, CAC: coronary artery calcium, CI: confidence interval, SBP: systolic blood pressure, SCAPIS: Swedish CArdioPulmonary bioImage Study.

© 2025 Herraiz-Adillo Á et al. *JAMA Cardiology*.

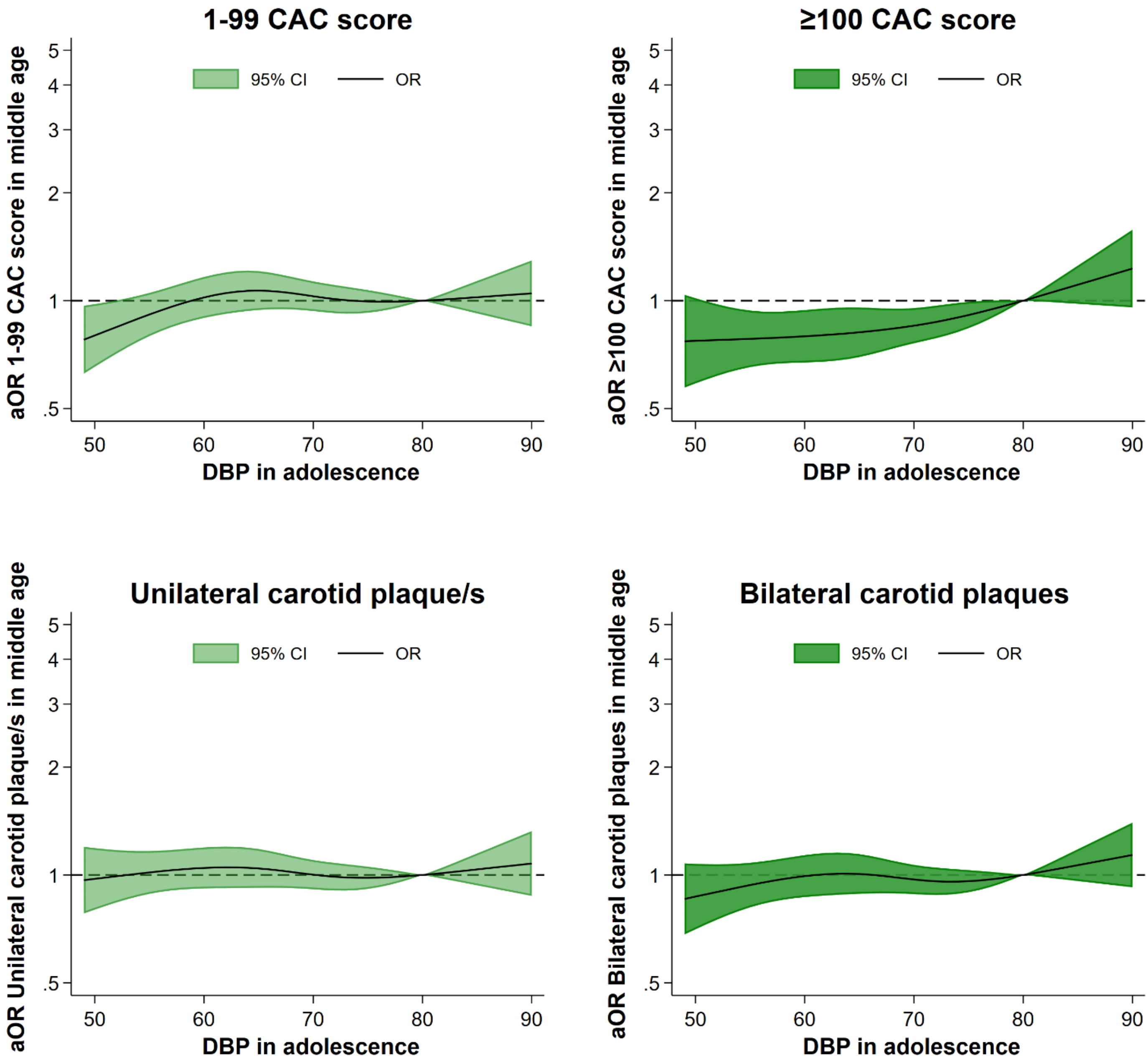

eFigure 16. Associations Across Restricted Cubic Splines of Diastolic Blood Pressure in Adolescence With CAC Score and Carotid Plaques in Middle Age

Multinomial logistic models are adjusted for age at conscription, site in conscription, year of conscription, BMI at conscription, smoking duration at conscription, age at SCAPIS, site in SCAPIS and educational level at SCAPIS. X-axes are trimmed to depict the associations for the 1st to 99th percentile of diastolic blood pressure values. Reference is set at 80 mmHg.

aOR: adjusted odds ratio, BMI: body mass index, CAC: coronary artery calcium, CI: confidence interval, DBP: diastolic blood pressure, SCAPIS: Swedish CArdioPulmonary bioImage Study.

© 2025 Herraiz-Adillo Á et al. *JAMA Cardiology*.

eReferences.

1. Ludvigsson JF, Berglind D, Sundquist K, Sundström J, Tynelius P, Neovius M. The Swedish military conscription register: opportunities for its use in medical research. *Eur J Epidemiol*. 2022;37(7):767-777. doi:10.1007/s10654-022-00887-0

2. Jones DW, Ferdinand KC, Taler SJ, et al. 2025 AHA/ACC/AANP/AAPA/ABC/ACCP/ACPM/AGS/AMA/ASPC/NMA/PCNA/SGIM Guideline for the Prevention, Detection, Evaluation and Management of High Blood Pressure in Adults: A Report of the American College of Cardiology/American Heart Association Joint Committee. *Hypertens (Dallas, Tex 1979)*. Published online August 2025. doi:10.1161/HYP.0000000000000249

3. McEvoy JW, McCarthy CP, Bruno RM, et al. 2024 ESC Guidelines for the management of elevated blood pressure and hypertension. *Eur Heart J*. 2024;45(38):3912-4018. doi:10.1093/eurheartj/ehae178

4. Sundström J, Neovius M, Tynelius P, Rasmussen F. Association of blood pressure in late adolescence with subsequent mortality: cohort study of Swedish male conscripts. *BMJ*. 2011;342:d643. doi:10.1136/bmj.d643

5. Bergström G, Berglund G, Blomberg A, et al. The Swedish CArdioPulmonary BioImage Study: Objectives and design. *J Intern Med*. 2015;278(6):645-659. doi:10.1111/joim.12384

6. Raff GL, Chair, Abidov A, et al. SCCT guidelines for the interpretation and reporting of coronary computed tomographic angiography. *J Cardiovasc Comput Tomogr*. 2009;3(2):122-136. doi:10.1016/j.jcct.2009.01.001

7. Bergström G, Persson M, Adiels M, et al. Prevalence of Subclinical Coronary Artery Atherosclerosis in the General Population. *Circulation*. 2021;144(12):916-929. doi:10.1161/CIRCULATIONAHA.121.055340

8. Ayoub C, Erthal F, Abdelsalam MA, et al. Prognostic value of segment involvement score compared to other measures of coronary atherosclerosis by computed tomography: A systematic review and meta-analysis. *J Cardiovasc Comput Tomogr*. 2017;11(4):258-267. doi:10.1016/j.jcct.2017.05.001

9. McCollough CH, Ulzheimer S, Halliburton SS, Shanneik K, White RD, Kalender WA. Coronary artery calcium: a multi-institutional, multimanufacturer international standard for quantification at cardiac CT. *Radiology*. 2007;243(2):527-538. doi:10.1148/radiol.2432050808

10. Agatston AS, Janowitz WR, Hildner FJ, Zusmer NR, Viamonte MJ, Detrano R. Quantification of coronary artery calcium using ultrafast computed tomography. *J Am Coll Cardiol*. 1990;15(4):827-832. doi:10.1016/0735-1097(90)90282-t

11. Touboul PJ, Hennerici MG, Meairs S, et al. Mannheim carotid intima-media thickness and plaque consensus (2004-2006-2011). An update on behalf of the advisory board of the 3rd, 4th and 5th watching the risk symposia, at the 13th, 15th and 20th European Stroke Conferences, Mannheim, Germany, 2004, . *Cerebrovasc Dis*. 2012;34(4):290-296. doi:10.1159/000343145

12. Herraiz-Adillo Á, Ahlqvist VH, Higueras-Fresnillo S, et al. Life's Essential 8 and carotid artery plaques: the Swedish cardiopulmonary bioimage study. *Front Cardiovasc Med*. 2023;10:1173550. doi:10.3389/fcvm.2023.1173550

13. Herraiz-Adillo Á, Ahlqvist V, Higueras-Fresnillo S, et al. Physical fitness in male adolescents and atherosclerosis in middle age: a population-based cohort study Br J Sports Med Epub ahead of print:30-01-2024. doi:10.1136/ bjsports-2023-107663.

14. Sederholm Lawesson S, Swahn E, Pihlsgård M, et al. Association Between History of Adverse Pregnancy Outcomes and Coronary Artery Disease Assessed by Coronary Computed Tomography Angiography. *JAMA*. 2023;329(5):393-404. doi:10.1001/jama.2022.24093

15. Textor J, van der Zander B, Gilthorpe MS, Liskiewicz M, Ellison GT. Robust causal inference using directed acyclic graphs: the R package "dagitty". *Int J Epidemiol*. 2016;45(6):1887-1894. doi:10.1093/ije/dyw341

© 2025 Herraiz-Adillo Á et al. *JAMA Cardiology*.
