# Supplementary material for: Blood Pressure in Adolescence and Atherosclerosis in Middle Age
Source: JAMA Cardiol. 2025 Nov 19;11(1):14–24. doi: 10.1001/jamacardio.2025.4271 (PMC12631567; doi:10.1001/jamacardio.2025.4271)
Supplement: Supplement 2. — Data Sharing Statement [file jamacardiol-e254271-s002.pdf]

Data Sharing Statement

Herraiz-Adillo. Blood Pressure in Adolescence and Atherosclerosis in Middle Age. *JAMA Cardiol*. Published November 19, 2025. doi:10.1001/jamacardio.2025.4271

Data
Data available: No

Additional Information
Explanation for why data not available: The data underlying this article cannot be shared publicly due to legal restrictions and to safeguard the privacy of study participants. However, those interested can obtain information on how to access the data in accordance with Swedish legislation by reaching out to the study organization at www.scapis.org.
